# Leveraging a novel NFT-enabled blockchain architecture for the authentication of IoT assets in smart cities

**DOI:** 10.1038/s41598-023-45212-1

**Published:** 2023-11-13

**Authors:** Usman Khalil, Owais Ahmed Malik, Ong Wee Hong, Mueen Uddin

**Affiliations:** 1 School of Digital Science, Universiti Brunei Darussalam, Jalan Tungku Link, Gadong, BE1410 Brunei Darussalam; 2 Institute of Applied Data Analytics, Universiti Brunei Darussalam, Jalan Tungku Link, Gadong, BE1410 Brunei Darussalam; 3 College of Computing and Information Technology, University of Doha for Science and Technology, 24449 Doha, Qatar

**Keywords:** Computer science, Statistics

## Abstract

The concept of smart city architecture requires a comprehensive solution that can combine real-time response applications for cyber-physical systems. However, the architecture faces challenges that can obstruct the operations in terms of systems, processes, and data flow as far as the breach risk is concerned. Though the field has been researched with the existence of centralized and distributed architectures to support smart cities. Research gaps regarding security concerns, platform assistance, and resource management continue to persist. This research article presents a novel blockchain-based architecture that proposes expansion in the non-fungible tokens (NFTs) to cater to the expansion of IoT-enabled smart assets. It enables NFTs to employ fog computing for all users and smart devices connected to a fog node in a cyber-physical system. The proposed expansion suggested in Non-Fungible Tokens (NFTs) for IoT assets representation in a cyber-physical system, provides devices and user identification and authentication functionality. The proposed NFT architecture has been designed to provide a smart city solution for cyber-physical systems that ensures robust security features (such as CIA) by introducing new attributes and functions for Owner, User, Fog, and IoT device/s authentication. The validation and rigor of the security services, efficiency, and latency have been achieved by deployments on private and public ledgers. The efficiency, and cost-effectiveness of the suggested functions and components have been evaluated in terms of evaluation cost and time complexity which resulted in promising results, obtained and validated on a testnet. The evaluation cost for the devised mint component was approximately 81%, and devised *approve()* was approximately 23% more efficient than other solutions.

## Introduction

A decentralized smart city, in the context of Web3, is an innovative urban concept that harnesses the power of blockchain technology to improve city operations and enhance the quality of life for its inhabitants. Decentralized technologies, such as blockchain, smart contracts, and decentralized applications (dApps), enable a decentralized smart city to provide greater transparency, security, and efficiency in managing urban resources like energy, water, and waste.

This approach has the potential to foster a more democratic system of governance, where residents can have more input into how their city is run through decentralized voting systems, resulting in a more equitable and democratic approach to urban development^1^. Another important aspect of a decentralized smart city considering the Web3 framework is the increased security and privacy of citizen data. By utilizing decentralized data storage and management systems, residents can have greater control over their data, ensuring that it is not exploited or misused by government or private entities.

Hence, various intricate privacy and security measures have been implemented in cyber-physical systems (CPSs) at the industry level^2^. One such popular example is the Distributed Control systems (DCS). The Internet of Things (IoT) networks further allocate the data to the cloud, fog, and edge layer for processing at different levels following the IoT paradigms.

**Figure 1:**
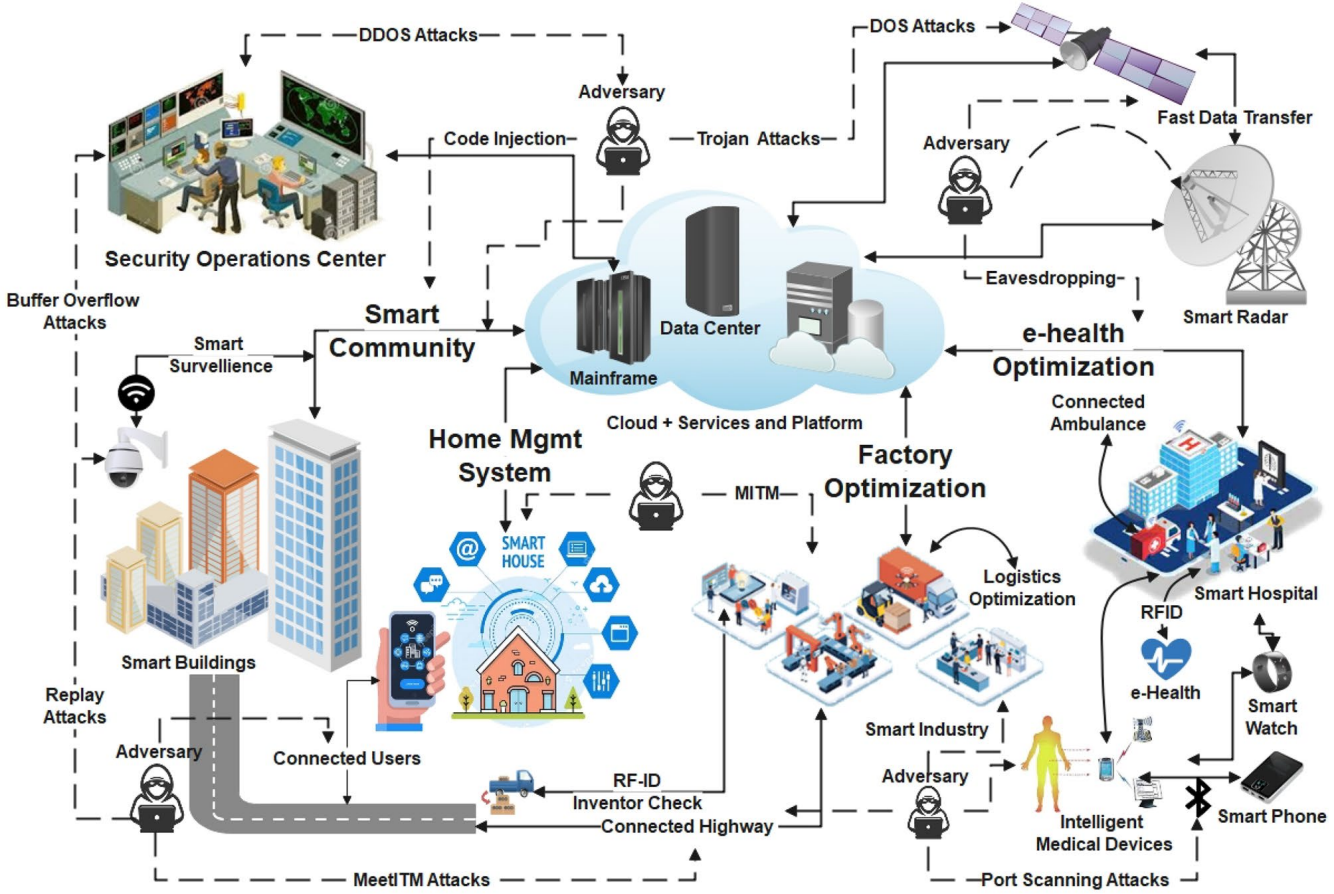
Broad-spectrum of smart city architecture and associated risk factors.

Figure 1 presents the smart city’s generalized architecture, depicting different CPSs working in different fields such as smart homes, smart grids, smart health monitoring, smart vehicles (UAVs—Unmanned Air Vehicles, UGVs—Unmanned Ground Vehicles), process control, oil, and gas distribution, transportation systems, etc. It utilizes cloud computing as a platform-based service model for data access, storage, analysis, and network to centralized data centers and IP networks^3^.

CPSs heavily depend on the extreme brim of the network that contains the edge nodes. These edge nodes provide limited resources regarding their data collection, storage, and processing efficiency while the IoT networks in CPSs provide a favorable environment for malicious actors for personal gains as shown in Fig. 1. Also, smart cities utilize technologies like software-defined networking (SDN), cloud computing (CC), and fog computing, inheriting the current threats in those arenas^4^.

### Blockchain tokenization

**Figure 2:**
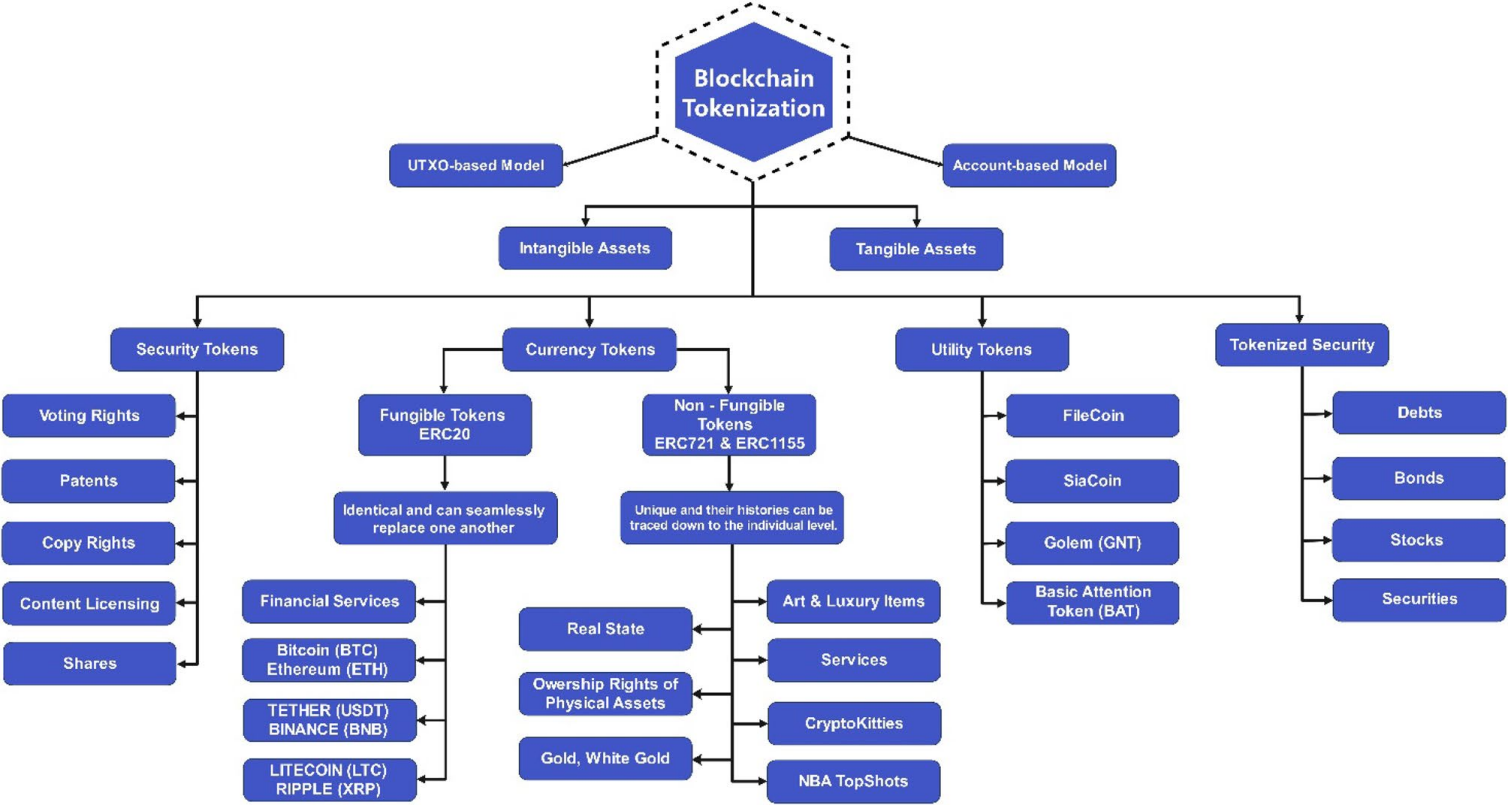
Blockchain tokenization.

The utilization of blockchain (BC)-based tokenization has emerged as a promising solution for asset identification and authentication mechanisms in smart city architecture. Since 2018, Token creation has gained immense popularity with a huge count of Initial Coin-Offering (ICOs) and Security Token Offering (STOs), which raised nearly $20bn^5^. This has led to the widespread recognition of the concept of tokens. Tokenization in blockchain introduces the idea of a digital representation of an asset on the blockchain, commonly referred to as a "programmable asset". There are two models in BC tokenization for transferring values using smart contracts i.e., the UTXO-based (Unspent Transaction Output) and the Account-based model^6^. Further BC tokenization presents different types of tokens, tangible and intangible, as depicted in Fig. 2. Among the different types of tokens, Security tokens have been utilized for voting rights, patents, copyrights, etc., and tokenized securities for debts, bonds, stocks, and securities. Utility tokens have been utilized for Filecoin, SiaCoin, Golem network, etc., and Currency tokens have been widely deployed to represent fungible and non-fungible assets^7^. BC offers tokenization mechanisms that are algorithms posted as a smart contract on a blockchain.

In the case of Non-Fungible Token/s (NFT/s), the ownership presents physical or digital assets, including physical property, virtual collectibles, and negative-value assets. Although NFTs are categorized as currency tokens, they can be used for specific purposes, such as the Multi Token Standard (ERC-1155), which allows fungible and non-fungible tokens to be combined in the same token. Some standards support royalty payments (EIP-2981)^8^ and mortgage/rental functions (EIP-2615)^9^, as shown in Fig. 2.

### NFTs for assets digitization

**Figure 3:**
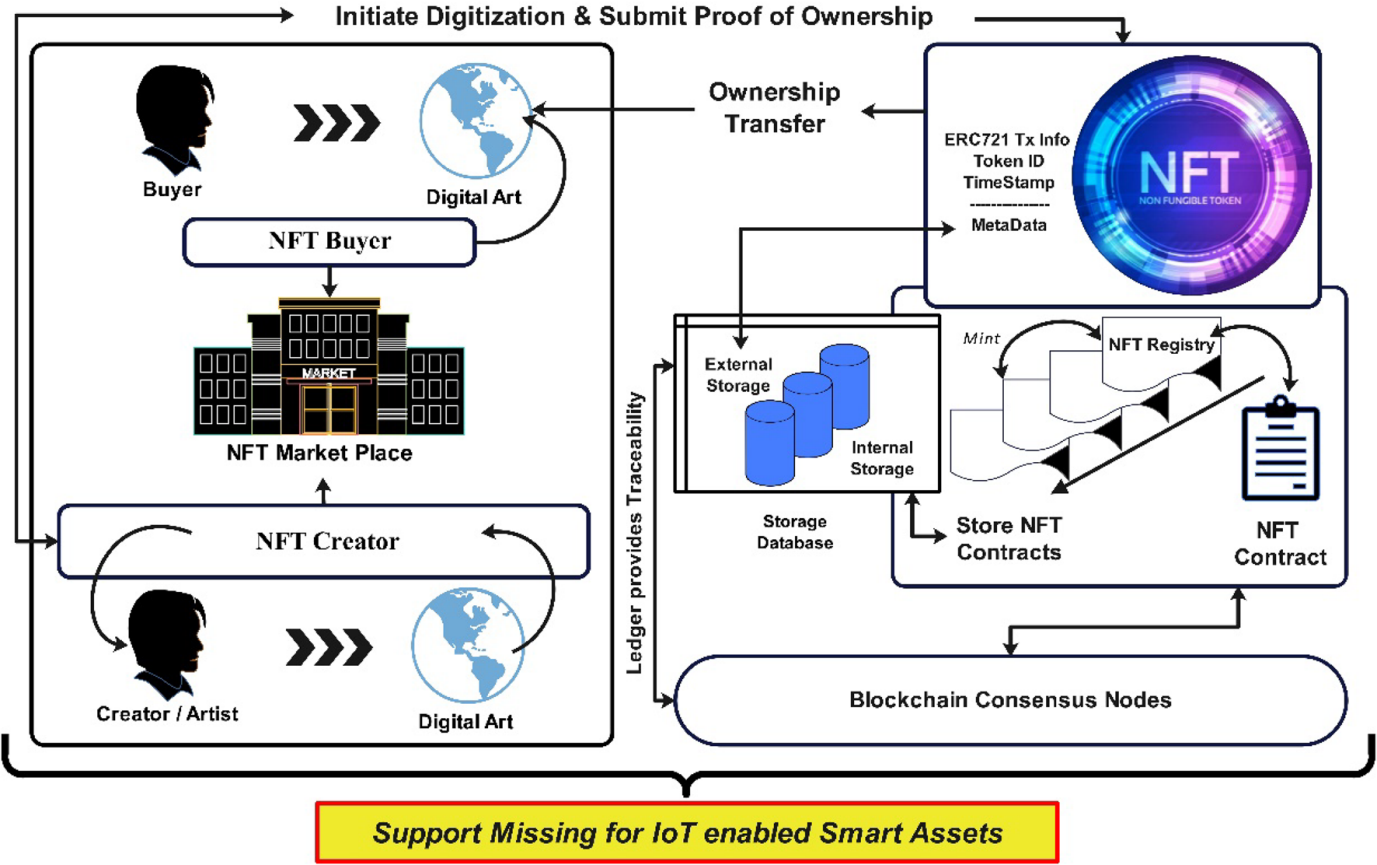
Generalized NFTs architecture.

Any asset linked to a distinctive cryptographic record refers to an NFT, usually a piece of art and luxury item, services in terms of music, real estate, collectibles, or another presumed valuable object as shown in Fig. 2. The asset refers to any physical asset that is a record maintained in the underlying distributed ledger and can be traded through transactions. These records can be bought sold and traded through digital wallets (such as Guarda, MetaMask, Exodus, and Coinbase to name a few^10^) in the form of tokens whose ownership can be claimed upon successful purchase by an NFT Creator/seller. Figure 3 depicts the generalized architecture of an NFT architecture in view of the NFT’s well-known project of CryptoPunks. It comprises two roles, i.e., owner and buyer. To digitize a resource, the owner checks the file, title, and description accuracy to generate the NFT. If the correct details are found, the raw data is digitized into a proper format through ERC721 standard-defined functions in the smart contract (SC)^11^. It is important to note here that the ERC721 standard for NFT lacks the ability to support the IoT-enabled smart assets in their current state.

The ERC721 standard functions in the NFT smart contract process the creator/owner’s request, which stores the raw data in a database external to the BC. However, the owner can also store the raw data in the internal blockchain database, which would not infer the execution cost for posting the transactions. Once the raw records are stored in the internal blockchain databank, the owner signs the transaction, including the NFT data hash. It is then sent to the SC, and stored in the NFT registry, as depicted in Fig. 3. Since NFTs are developed and deployed on BC, the blockchain consensus is of much importance in the NFT architecture.

At this point, the smart contract from the NFT registry receives the NFT data transaction. It is ready for the minting and trading process. Here logic in the form of transactions is processed to the consensus nodes in a P2P network to attain consensus for privacy. Once the logic of the ERC721 Token Standard triggers, the NFT data is minted. The transaction posting confirms the minting process, which can be traced at any time with a unique blockchain address providing traceability of the digital assets on BC. The ledger provides the traceability of NFTs, which provides a tangibly defined “digital impression” as a unique identifier. The NFT buyers can transfer the proof of ownership after an approved agreement with the NFT Creator.

### Problems associated with the cutting-edge proposed authentication mechanisms

The literature survey presents the current cutting-edge security authentication mechanism for IoT assets in a distributed CPS architecture. The assessment summary of the proposed authentication schemes is presented below.

- The verification of these smart contracts (SC) may face challenges since IoT-enabled smart devices may be inconsistent.
  - SCs in proposed solutions are typically not designed with the heterogeneity and constraints of IoT-enabled smart devices in mind, particularly in the context of the smart city concept.
  - The use of functions and events in smart contracts (SCs) allows for faster implementation of actuation mechanisms in IoT-enabled smart devices.
  - The deployment of smart contracts that include defined authentication functions can enhance security, thus necessitating the consideration of authentication schemes involving smart contracts/decentralized apps (dApps).
- By default, the firmware of IoT-enabled smart devices does not possess a complete security mechanism, which leads to security vulnerabilities from the manufacturer's standpoint.
  - Especially authentication, access control schemes, and firmware updates are commonly found unattended, posing these assets’ exploitation. In particular, authentication mechanisms, access control schemes, and firmware updates are often neglected, leaving these assets vulnerable to exploitation.
  - In order to alleviate the issues related to authentication and access control based on communication and computational costs, new encryption schemes such as SHAIII, which are both strong and lightweight, can be considered.
- Most of the proposed mechanisms have been implemented on the Ethereum platform, which employs the conventional Proof of Work (PoW) consensus mechanism. Although Ethereum supports the development and deployment of public, private, and hybrid blockchains, it also allows decentralized applications (dApps) to perform functions as needed. Numerous blockchain platforms, including Hyperledger Besu^12^, Hyperledger Fabric^13^, Solana^14^, etc., feature more efficient consensus mechanisms compared to the traditional Proof of Work (PoW) consensus mechanism employed by Ethereum, which could be used to develop smart contract-based solutions with improved performance.
  - The implementation of blockchain-based solutions for smart city infrastructure should consider the efficiency and security of the underlying consensus mechanism employed. Although the traditional Proof of Work (PoW) consensus mechanism in Ethereum has been commonly used in proposed solutions, other platforms like Hyperledger Besu, Hyperledger Fabric, and Solana offer more efficient alternatives, including IBFT, IBFT 2.0, and Clique. These consensus mechanisms should ensure robust fault tolerance, decentralization, stability, and high-level security and authentication stability of IoT-enabled smart devices to support the smart city infrastructure. The effectiveness of these schemes has been evaluated based on security services for collaborative authentication, decentralization, and stability. The analysis reveals that most issues are related to access control and data anonymity.
  - The proposed mechanisms in recent studies aim to achieve decentralization by employing blockchain but they lack sufficient security and reliability. As a result, it is imperative to develop a consensus mechanism that is not only robust but also reliable to address the security issues in the blockchain.
- In recent times, Physically Unclonable Functions (PUFs) have emerged as a preferred option considering Trusted Platform Modules (TPMs) for identifying devices in blockchain-based solutions.
  - Since PUFs result from hardware modification, they come with the cost of modifying the device properties and adding manufacturing costs to the budgets, making it hard to develop to implement in smart city scenarios.
  - As there will be billions of devices connected to the internet, in the case of smart cities, the manufacturing costs to develop PUFs would not be suitable for Governments and businesses to consider such implementation.
- In a recent research work^15^, the utilization of non-fungible tokens (NFTs) has been proposed to represent unique assets and their ownership using a unique identifier. However, in this study, these tokens were utilized to bind IoT assets physically using PUFs.
  - The representation of assets also incorporates authentication mechanisms that rely on PUF to capture the physical characteristics of devices. These mechanisms leverage the devices' private keys and BCA addresses to identify and represent them. The mechanism, however, has not been designed to cater to a complete set of security services (CIA & AAA).

The approach relies on Physical Unclonable Functions (PUF) as an additional hardware component, which must be included by the manufacturer. Based on issues associated with the solutions in the literature, the research gap has been presented which leads to the methodology of the proposed architecture.

### Research gap

Since the blockchain-based architectures to depict admin, users, edge, and fog devices utilizing non-fungible tokens (*NFTs*) in the literature are explicitly lacking, the proposed NFT-enabled expansion utilizes newly defined attributes for representation from a software perspective leaving off the need to update the customer premises equipment (CPE) hardware. The resource-constraint nature of the edge nodes (i.e., low processing power, low data storage capabilities, low computational resources, etc.) concerning the digital representation, implementation, and authentication aspects of smart IoT assets in a distributed architecture has been explored. It prompts the formulation of the following research questions:

1. How to develop and deploy Web3 infrastructure to attain decentralization for assets in cyber-physical systems for a smart city?
2. How does a smart contract provide an efficient solution utilizing blockchain tokenization (NFTs)?
3. How to describe the authentication mechanism of assets in cyber-physical systems for a smart city concept using blockchain tokenization over Web3 infrastructure?
4. Is it possible to utilize NFTs without the hardware modification of CPE?
5. How to evaluate and utilize the potential of smart contracts to attain robust security?

Based on the research questions, the contributions of the study have been presented. The subsequent sections of this work embark on an in-depth exploration, driven by the overarching aim of fulfilling the research objectives.

The literature review discusses a proposal of ERC721 in^15^ which involves a hardware elevation from the manufacturer resulting in manufacturing costs. However, the proposal only takes into account the IoT device-level security perspective and does not present the architecture for cyber-physical systems. Also, it comes with a few notable issues such as, in case of device malfunction, the association of the NFT with hardware properties may cause a system failure. Furthermore, IoT assets with hardware upgrades have extended startup time, resulting in latency issues, such as initializing the Bootloader in the main System on Chip's (SoC) internal one-time programmable memory. Since the Bootloader serves as the device's Root of Trust, it cannot be modified. Furthermore, the on-chip Static-RAM, which is also regarded as an SRAM PUF, cannot be modified, but it raises notable concerns regarding time complexity, computational complexity, and latency problems.

As opposed to that, non-fungible tokens for cyber-physical systems depict weak prevalence due to the need to develop interchangeable and novel components to handle the transaction volume related to the digitization of IoT assets and data generated by them. Novel modules are inevitable for non-fungible currency tokens to handle fractional ownership and divisible units. This observation highlights a research gap, emphasizing that although non-fungible currency tokens are less common, their utilization as a representation in blockchain tokenization would require novel considerations to employ their unique characteristics and divisibility challenges in cyber-physical systems. Hence, the NFT functionality has been expanded to be employed keeping in view the ERC721 standard for smart assets. Despite the development of major categories, the ERC721 tokens do not define any of the attributes of the smart city infrastructure where assets can be identified by a public key and transact uniquely by the identifiable tokens in their original form.

These unique characteristics through divisibility can be achieved by dependencies like the smart contract that provides a function-based interface to build non-fungible tokens (NFTs) on decentralized networks. According to the set objectives, smart city infrastructure for cyber-physical systems based on the distributed network must be explored to provide security for nodes at the sensing and application layers. The article proceeds to elucidate the design elements of the proof of concept as initially detailed in^16,17^. The details are further explored in the upcoming sections.

### Contributions

As depicted in Fig. 2 blockchain tokenization has been utilized in many domains whereas the NFT architecture in Fig. 3 shows the adaptable tokenization of assets, which offers security comparable to that of cryptocurrency and has the potential for extensive token usage. Nonetheless, the standard does not have the capability of defining attributes specifically for smart city applications, also the evidence of such attributes is missing in the available literature. Thus, a novel NFT-based blockchain architecture has been proposed through which the smart city applications for underlying cyber-physical systems can be developed and deployed leading to the contributions made in this research.

In light of the details mentioned in earlier sections for fog computing, blockchain, and the utilization of blockchain tokenization for user and device authentication based on decentralized architectures provides a new dimension, yet provokes new challenges. The research outlined in this article focuses on the following points, which represent the major contributions of this study.

- Our work proposes an innovative NFT-based authentication structure encompassing Owners, Users, fog, and IoT nodes. This architecture aims to digitize IoT assets within smart city infrastructure, enhancing security and accessibility.
- We pioneer a unique approach for integrating IoT-enabled smart devices through tokenization within a decentralized IoT framework. Notably, this mechanism operates independently of centralized entities like Cloud Services, fostering a more autonomous infrastructure.
- Leveraging Externally Owned Addresses (EOA) in blockchain architecture, we establish NFTs as digital representations of smart devices. This adaptation addresses a deficiency in the existing ERC721 standard, allowing for a more robust smart device portrayal.
- Finally, the proposed architecture presented in this research paper centers on the software-based digital representation and authentication of IoT-enabled smart assets. It eliminates the need for any additional hardware upgrades from the manufacturer, such as Physical Unclonable Functions (PUF).

The rigor of the security services, efficiency, and latency have been achieved by evaluations of deployments on private and public ledgers in line with the execution of the contributions in this proof of concept. The paper structure has been ordered as follows. The Literature Survey section discusses the literature review of blockchain-based authentication mechanisms with security services and associated problems in smart city architecture. The Methodology section presents the methodology of the novel NFT architecture along with a stepwise working methodology while the section Design and Implementation discusses the design and implementation aspects of the proposed NFT architecture for the IoT assets such as user, fog, and smart devices authentication over Hyper Ledger Besu, Goerli Testnet, and related architectures. Section Results and discussion presents the implementation and validation of devised components and costs in smart city architecture with results and proof of concept. Finally, a succinct summary concludes the research article.

## Literature survey

The review of the existing literature in this article has emphasized the authentication schemes implemented on blockchain and the digital representation of IoT assets. A comparative analysis has been presented to examine the security challenges associated with these architectures keeping in view the IoT-enabled smart assets within a smart city framework.

### Authentication mechanisms on decentralized networks

A distributed authentication mechanism for IoT assets has been proposed in^18^. The mechanism leverages the benefits of fog computing and public blockchain technologies. The mechanism consists of a device-to-device (D2D) communication phase for device communication in and out of the system, and access control for fog and IoT devices, achieved through the use of the Elliptic Curve Digital Signature Algorithm (ECDSA) for key generation. The proposed mechanism was tested for various security requirements, including confidentiality, integrity, identification, non-repudiation, authentication, and mutual authentication.

In^16^, a framework called *Blockchain of Things Sentry* is proposed, which integrates the benefits of blockchain and IoT networks, and enhances network security by analyzing the device's network traffic flow patterns obtained from data storage on the blockchain. The framework aims to maintain the lightweight nature of IoT devices, which often cannot meet the computationally intensive requirements of blockchain-based security models. The framework includes BCoT Gateways—blockchain nodes that employ an IoT device security module managed via a smart contract (SC).

In their work, a unique approach has been introduced for feature selection in machine learning using the maximum information coefficient (MIC) to evaluate the discrimination of IoT assets. Their approach captures IoT device traffic from the network layer and transmits it as a traffic flow feature to the smart contract through blockchain transactions. Subsequently, the smart contract identifies the device and performs relevant operations automatically after the transactions are recorded on the blockchain.

In^17^, a decentralized authentication modeling architecture called *BlockAuth*, utilizing blockchain technology, is proposed. The architecture considers edge devices as nodes forming a blockchain network, where various authentication methods, such as word identification, certificate-based, biotechnology-based, and token-based, can be employed to meet high-security requirements in IoT and edge environments. The architecture leverages blockchain consensus and SC features. The authors in^19^ a cost-effective and lightweight tool called *SmartEdge* for managing compute resources in edge computing. This tool is based on a public blockchain that employs the smart contract. The design follows three key steps, which the authors describe in detail. The authors also provide a traditional representation of the device, while evaluating the performance of the tool in terms of cost-effective delay in executing a job and transaction cost, which are insignificant compared to their value.

The authors in^20^ proposed a novel architecture for *Distributed Anonymous Multi-Factor Authentication* for resistance to offline attacks and to incorporate blockchain to enhance usability. As a result, the authentication process no longer depends on a trusted third party and requires no interaction with the identity provider, providing improved security and convenience. To evaluate the proposed mechanism, the authors utilized Namecoin, a public ledger blockchain that offers the ability to register names and store associated values in a secure and distributed shared database.

*BCTrust* as a framework has been proposed in^21^ for implementing an authentication mechanism based on blockchain, which is suitable for devices with limited resources, such as storage, computation, and energy consumption. The authors deployed the mechanism using the public blockchain Ethereum, which provides robustness through its distributed ledger technology and the absence of a central authority for signing contracts, known as smart contracts.

A novel approach for user authentication in IoT devices has been presented in^22^, which involves the use of blockchain-enabled fog nodes. The proposed mechanism employs smart contracts to authenticate users seeking access to IoT devices. The proposed system employs fog nodes to maintain, register, and manage IoT devices, admins, and end-users while providing scalability to the system. This approach relieves IoT devices from carrying out heavy computations that involve tasks such as authentication and communication with the public blockchain. In order to enable authentication of IoT devices at scale, a distributed system design based on public blockchain technology has been proposed with implementation carried out using Ethereum smart contracts. The smart contract proposed in this study is responsible for implementing the authentication functionality necessary for the addition of both end-users and IoT assets. An administrator oversees the overall functionalities and operations of the authentication mechanism.

Authors in^23^ address a fundamental challenge in IoT security decentralized authentication through a novel blockchain-based mechanism. The authors introduce an innovative authentication system tailored for distributed and heterogeneous IoT networks. It merges several key concepts, such as blockchain technology, environmental signatures, and the Received Signal Strength Indicator (RSSI), into a unified approach. A noteworthy feature of this authentication mechanism is its support for both forward and backward secrecy, facilitated by the incorporation of blockchain and elliptic curve cryptography. However, the paper acknowledges the necessity for further research to validate the system's performance. Specifically, it highlights the need to assess resource utilization and the time required for validation, recognizing the importance of these aspects for practical implementation.

Research in^24^, suggests a blockchain-based architecture for trust and authentication in a decentralized network, utilizing a public key encryption system. A model called the *Web of Things* enhances interoperability and transparency while minimizing the chain of trust by leveraging web technologies. To establish a scalable and decentralized PKI specifically for IoT-enabled smart devices, a hybrid WoT model with the web-3 authentication and authorization framework has been proposed.

### IoT-enabled smart device description

The use of physically integrated chips (ICs) embedded in IoT assets has been adopted to represent smart devices to alleviate the risk of physical attacks on smart assets. This unique feature provides an added layer of security that eliminates the possibility of physical tampering by adversaries such as impersonation and side-channel attacks. On the other hand, ICs such as Trusted Platform Modules (TPM) utilize a previous platform hash due to the fact that these platform hashes are both signed and timestamped which can be utilized by IoT assets. Nevertheless, the integration of this security aspect results in considerable communication and latency overhead, thereby impeding the application of IoT-enabled smart devices in Cyber-Physical Systems (CPSs), particularly in time-sensitive scenarios.

#### Smart device representation using distributed architectures

*xDBAuth *as a decentralized blockchain-based framework has been proposed in^25^. The authors suggested the shortcomings of single trusted third-party approaches. Local and global smart contracts have been incorporated for permissioning and access control requests. The Proof-of-Authenticity/Integrity (PoAI) for authentication and validation has been achieved through a Trusted Platform Module (TPM), which is essentially a microcontroller capable of securely storing critical artifacts necessary for platform authentication. The authors assume that the user or IoT device cannot utilize a previous platform hash because these platform hashes are both signed and timestamped by the TPM.

Research in^26^ suggests a blockchain-based solution aimed at authenticating users securely for IoT device access. The approach is designed to address the limitations prevalent in existing authentication methods, offering an improved alternative. Utilizing Ethereum smart contracts, the blockchain-based solution ensures the creation of tamper-proof records while the solution achieves decentralization. Additionally, the authors explore the monetization aspects pertinent to IoT devices and their data which may contemplate a system where usage is compensated through cryptocurrency tokens, such as Ether.

In a recent research article, a novel platform has been suggested by the authors that leverages blockchain technology for IoT device authentication, security services, and data privacy using smart contracts. The study involved implementing this platform on Hyperledger Fabric, which achieved a throughput rate of 35 transactions per second (TPS) while also enabling IC traceability. The proposed approach described in the study utilizes physically unclonable functions (PUFs) as a defined function on integrated chips (ICs) to integrate the authentication mechanism factors. Furthermore, the hardware of IoT devices was customized specifically to optimize the performance of the blockchain. This proposed method has the potential to offer an effective solution to address security concerns in the IoT environment^27^.

The authors established a trust base controlled by smart contracts to ensure that users have private access to their IoT devices and data. They devised a mechanism for the remote configuration of IC features via smart contracts, enabling secure and repeated programming of an IC.

The authors in^15^, introduced a unique approach for asset representation using non-fungible tokens (NFTs) as a possession identifier of an owner. The proposed method involves binding the NFT to its respective IoT device in a smart manner. In addition, the proposed approach includes a specification for authentication mechanisms that rely on Physical Unclonable Functions (PUFs) to capture the unique physical characteristics of the devices. These PUFs are then used to establish the device's identity and to generate its private key and corresponding blockchain address.

The approach utilizes non-fungible tokens (NFTs) to represent assets by assigning a unique identifier as proof of ownership. The NFTs are linked to specific IoT devices and assigned a blockchain account (BCA) address to facilitate participation in blockchain transactions. Secure communication channels between the NFTs and their owners or users can be established, and they can operate dynamically in different modes associated with their token states. The proposed approach was implemented using ESP32-based IoT devices and the Ethereum blockchain. The ESP32's SRAM was utilized as a physically unclonable function (PUF) to enhance security.

### Comparison of NFT-based solutions for smart cities

In this comparative review, an analysis has been carried out among the recent NFT-based research. By examining these studies, we focus on the goal of gaining insights into the novel and diverse applications of non-fungible token (NFT) enabled solutions in the context of smart cities as shown in Table 1. It will help to identify potential areas of improvement and shed light on the research gap.

**Table 1:** Comparison of NFT-enabled Solutions with the proposed NFT-expansion.

Solution Description	Advantage	Drawbacks
NFT-based Asset Management—2021^15^	Utilizes NFTs for tracking and managing assets in smart cities, providing unique ownership and provenance information Propose components to extend the NFT to formulate its scope	Authentication mechanisms rely on hardware upgrades Hardware performance issues with startup time, resulting in latency issues Device Root of Trust cannot be altered SRAM cannot be modified which leads to computational complexity No support for security Services such as Confidentiality, Integrity, and Availability (CIA) has been provided
Connect2NFT: NFT-driven Urban planning—2022^28^	Integrates NFTs to facilitate community participation and decision-making in urban planning and development projects to secure user authentication	Framework utilizes NFT in its general form for users to associate social media accounts to authenticate owners The framework is dependent on the default NFT standard while security Services such as the CIA have not been taken care of
NFT-enabled IoT Security—2022^29^	Utilizes NFTs to secure Internet of Things (IoT) devices and networks, preventing unauthorized access and ensuring data integrity	Complete details of the system are missing The framework is dependent on default NFT components which leads to general implementation The framework is dependent on the default NFT standard for data integrity while security Services such as the CIA have not been taken care of
NFT-backed Tokenized Services—2022^24^	Implements NFTs to tokenize and trade healthcare services in smart cities, enabling efficient and transparent service exchange	A general association of healthcare assets (i.e., patient’s details, medicines, etc.) to the default NFT standard No divisibility of components to define IoT assets in a smart city context Framework security is dependent on the default NFT security mechanism while security services such as CIA have not been taken care of
NFT-Vehicle: NFT-based Digital Representation of the vehicle as a smart city asset—2023^30^	Implements NFTs to create a digital representation of vehicles as smart city assets, enabling real-time monitoring and analysis, and security in the form of associating ownership to the asset	NFT-Vehicle involves the general implementation of NFT for the associating stakeholders such as the manufacturer, owner, government, etc No architecture is defined for IoT assets in a smart city Implementation security is dependent on the default NFT security mechanism through its default ownership mechanism while security services such as the CIA have not been taken care of
NFT-driven Urban Governance-2022^31^	Integrates NFTs to facilitate transparent and accountable governance processes through the tracking and tracing of the Pharmaceutical Supply Chain for healthcare in smart cities	NFT deployment to associate pharmaceutical assets (medicine details) to the default NFT standard No divisibility of components to define IoT assets in a smart city context Implementation is dependent on the default NFT security mechanism while security services such as the CIA have not been taken care of

**Figure 4:**
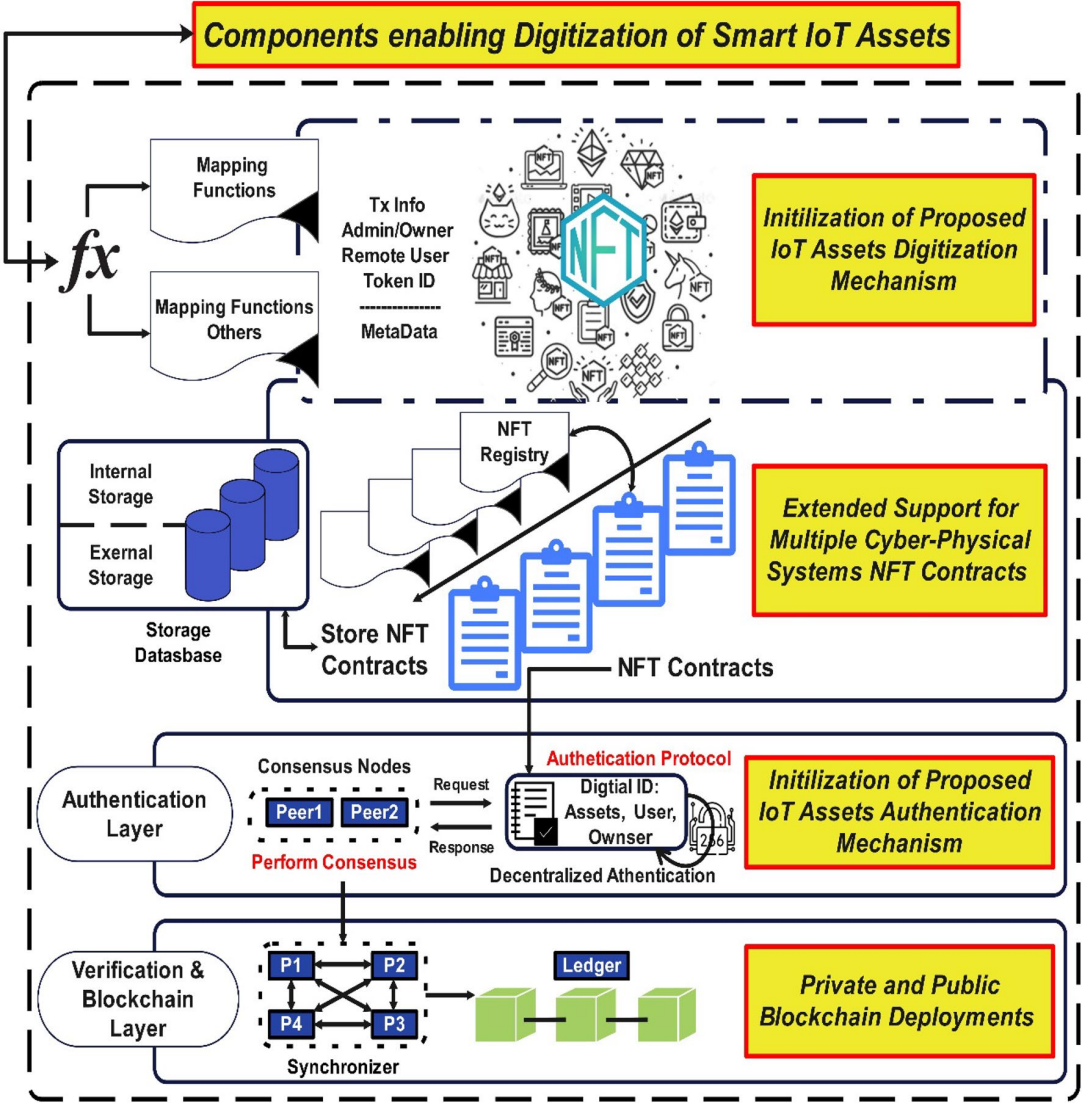
Methodology—the proposed NFT-enabled Architecture for the cyber-physical system.

As discussed in the previous section this is the only research in literature^15^ that proposes the extension in the current form of the erc721 tokens, however, fails to demonstrate a complete architecture of all IoT assets and their authentication mechanism. Also, the device requires a hardware upgrade for authentication mechanisms that rely on Physical Unclonable Function (PUF) to capture the unique physical characteristics of the devices.

The research in^28^ introduces a framework “*Connect2NFT*” for NFT-based urban planning that emphasizes community engagement and decision-making. The research highlights the potential of NFTs in fostering transparency and inclusivity in urban planning while the framework only utilizes NFT in its current form.

This study^29^ investigates the use of NFTs to enhance IoT security in smart cities. The researchers utilize an internally developed blockchain to store reputation values within their system. The research focuses on an identity-based encryption system while the complete implementation details of their system were not disclosed, which leads to ambiguities in system understanding.

The research explores the use and implementation of NFTs for healthcare product management^24^. The authors use the NFT functionality of digital certification to establish and maintain product ownership ensuring ownership integrity, seamless trading, and traceability. However, fails to demonstrate a complete architecture of IoT assets and their authentication mechanism-related expansion in NFTs.

*NFT-Vehicle* presented in this study^30^ is merely an effort to implement and demonstrate the applications and potentials of NFTs in one of the domains of smart city architecture. The author discusses the association of stakeholders such as the manufacturer, owner, government, etc. to its vehicle to enhance security in terms of ownership. However, no mechanism for security services such as Confidentiality, Integrity, and Availability (CIA) has been devised.

The authors in^31^ merely present the association of pharmaceutical supply chains to attain ownership security and data integrity by minting NFTs on the blockchain’s default security mechanism. It’s general NFT implementation that fails to demonstrate a complete architecture of IoT assets and their authentication mechanism-related expansion in NFTs. Apart from deployment, the mechanism for security services such as Confidentiality, Integrity, and Availability (CIA) has not been devised which raises security concerns.

The comparison in Table 1 among the NFT-enabled solutions makes it clear that the solutions have implemented the non-fungible tokens in its default form to attain digital identity while the security and privacy of these solutions purely depend on the NFT default mechanism. This can lead to security issues related to unauthorized access, data breaches, and compromised integrity of IoT-enabled smart assets and their digital representations. Secondly, none of the solutions propose components to define the IoT assets and authentication mechanism for enhanced security apart from the research in^15^ which focuses on device authentication mechanisms and does not offer a complete architecture.

On the contrary, the mechanism proposed in this research focuses on proposing expansion in the NFT standard through ERC721. The expansion enhanced the NFT's capability to establish a decentralized digital identity management system for IoT assets in a cyber-physical system. The study proposes a framework where NFTs are used to represent and authenticate unique digital identities, providing stakeholders with control over the data and enhancing security and privacy. By expanding NFTs, the proposed framework aims to provide users with unique ownership of planning decisions and assets, enhancing transparency and inclusivity in the urban planning domain.

## Methodology

The methodology employed in this research constitutes a comprehensive framework that outlines the systematic approach utilized to address the research contributions. It encompasses the carefully chosen methods, techniques, and tools employed to collect, analyze, and interpret the data, ensuring the credibility and rigor of the study. By explicating the methodology, this research aims to provide transparency and a clear understanding of the proposed NFT processes undertaken to arrive at meaningful evaluation in Results and discussion section through actionable insights.

### Proposed extended NFT standard for cyber-physical systems

The working methodology is innovative since a novel expansion in NFT standards has been presented for cyber-physical systems in smart cities. The expansion presents a standardized approach to deploying smart IoT asset representation by expanding non-fungible tokens (NFTs) compatibility and further devising authentication mechanisms through smart contracts. The proposed methodology is designed to implement a novel approach, as depicted in Fig. 4. The following objectives serve as essential expectations for this methodology:

- Expansion of ERC721 Protocol: Innovative components will be devised to extend the capabilities of the current ERC721 protocol, enabling it to cater to the digitization of smart IoT assets.
- IoT Assets Digitization: The proposed digitization mechanism for IoT assets will be initialized by incorporating relevant attributes as metadata, ensuring efficient representation and management
- Authentication Mechanism: A robust authentication mechanism will be introduced to add a layer of security for the IoT assets, safeguarding against unauthorized access.
- Cryptographic Primitives: To enhance the security of the authentication mechanism, a 3rd generation encryption protocol will be employed, ensuring data integrity and confidentiality.
- Deployment on Public and Private Blockchains: The methodology will encompass deployment on both public and private blockchain ledgers, facilitating monitoring and evaluation, and gaining valuable insights into its performance and effectiveness.

Through the fulfillment of these objectives, the proposed methodology aims to provide an innovative and secure approach for digitizing and authenticating IoT assets, contributing to the advancement of blockchain-based solutions in the context of IoT assets.

### Working mechanism for assets and IoT-enabled smart device authentication

A smart contract (SC) has been devised with the functionality of NFT/s for digitally declaring the IoT assets through proposed components that enable an association mechanism with the devised metadata of attributes to bind them with the Externally Owned Addresses (EOAs). The process triggers the initialization of asset digitization through which the assets will be associated with their respective proposed NFTs as shown in Fig. 4. Through this, the deployed smart contracts achieve the capability to interact with the resource constraint IoT assets based on the decentralized application (dApp) by newly defined NFT attributes for device representation operating at the edge of the sensing layer. The elaboration of the methodology's objectives, including the extension of the ERC721 Protocol, digitization of IoT assets, implementation of cryptographic primitives, and deployment on both Public and Private ledgers in the subsequent sections may further provide logic.

#### Extended support for multiple SCs

The architecture provides extended support for multiple smart contracts that enable the architecture to deploy multiple contracts for different CPSs. Once the SCs are deployed the NFT registry stores the transaction (Tx) volume and information, including details pertaining to the NFT-based externally owned accounts (EOAs) of the User, fog, IoT assets, proposed metadata, and TokenID through devised components to cater the expansion for IoT-enable smart assets. This storage mechanism is employed to effectively authenticate the relevant IoT assets. The internal storage is utilized to store the NFT registry. In contrast, the external storage is utilized to access the NFT registry externally by the users, as demonstrated.

Another important mapping aspect has been devised to map newly defined NFT attributes of users, fog nodes, and edge nodes with respective users and devices. It would help attain the security services for authenticating users with fog and edge devices in smart city architecture, i.e., confidentiality, integrity, authorization, and availability. Thus, the mechanism is not dependent on the default security mechanism through the NFT default ownership mechanism. Also, the IoT assets will not be dependent on supplementary hardware upgrades from the manufacturer, as seen in the case of^15,27^, i.e., Physical Unclonable Functions (*PUF*).

#### Authentication layer

The inclusion of an authentication layer in Fig. 4 shows a mechanism that aims to provide decentralized authentication and authorization for nodes connected to Cyber-Physical Systems (CPSs) in the context of a smart city. It is intended to serve as an authentication logic for a decentralized application. Specifically, the proposed architecture leverages the *SHAIII* encryption protocol to facilitate robust authentication of IoT assets. By utilizing an authenticated encryption system, the proposed architecture would benefit from faster hashing. Given that decentralized cryptosystems have been preferred for building solutions on blockchain technology due to the potential security vulnerabilities introduced by centralized systems, such as those involving trusted third-party (TTP) service providers.

Upon successful user authentication using the *mint* function in relation to the fog and IoT assets, the consensus mechanism is triggered, which is IBFT 2.0 in this case. The Tx is then shared with peers in the P2P network and synchronized with the appropriate group by synchronizer nodes before being recorded as an immutable transaction in the verification and blockchain layer. These posted transactions offer traceability through the use of unique identifying codes, which are associated with each proposed NFT. This digitization of assets allows for easy tracing over private and public ledgers.

#### Verification and blockchain layer

Verification and blockchain layer play a pivotal role as platforms based on Ethereum offer many more functions and logic for business models apart from cryptocurrency through smart contract implementation. The primary objective of the blockchain layer is to ensure the provision of security services, such as confidentiality, integrity, availability, authentication, authorization, and audit (CIA & AAA) to the users and CPE (i.e., sensors and actuators). Additionally, it aims to establish the identification of CPE within CPSs in a decentralized fashion in smart cities. The transaction (Tx) volume is handled by the deployment, which provides rigor traceability audibility, and immutability in a trustworthy manner. The creation of layer-two platforms triggered the invention of the public and private platforms whose architecture is supported and deployed on the Ethereum platform, such as Hyper Ledger Besu (HLB)^12^, Hyper Ledger Fabric (HLF)^13^, and public testnets such as Goerli^32^ to validate the results obtained in a test environment compared to the private deployment which may be helpful as it is a valuable tool for testing and implementing blockchain-based decentralized apps.

## Design and implementation

Smart contracts are utilized to create client-side applications that operate as decentralized apps (dApps) on top of the blockchain. These applications are typically built using the Solidity programming language, which shares similarities with JavaScript. The development, compilation, and deployment of the proposed mechanism as an extended NFT protocol was carried out using the Remix IDE (v0.23.3). As previously discussed NFTs are based on the Ethereum Request for Comment ERC-721, which defines a standard interface for wallet applications to operate with any NFT on Ethereum platforms. Unlike its fungible and interchangeable predecessor, ERC-20 tokens, ERC-721 tokens are unique to each assigned asset and therefore non-interchangeable. This distinctive property makes them an attractive option for extending their capabilities for smart devices (non-fungible).

The proposed NFT-based architecture was established in Solidity programming language on Remix IDE. The smart contracts were also deployed on Ethereum based public testnet stream (Goerli) at the address of the smart contract: 0 × 504C7FAb97AFb2642Bb00Fff8520AbA0857E3544 which is now publicly available for testing, and deployment purposes. The devised functions have been tested and yielded successful results. Additionally, the number of computational resources used, as measured by Gas consumption during the transaction, showed significant findings. A graphic representation has been given in "Validation of proposed NFT architecture" section in detail while the gas consumption of devised functions has been presented in "Efficiency performance" section.

The proposed NFT expansion employs NFTs to represent smart devices, with each device assigned a token_ID_ and an NFT-based Externally Owned Address (EOA) to serve as a resource owner. These NFTs provide both identity and uniqueness in attributes for assets, enabling them to be owned, transferred, and authorized for use. However, while NFTs offer a basic level of asset representation, additional attributes such as owner information, device functionality, and remote user details are not explicitly incorporated. To address this limitation, a novel NFT-based architecture is proposed that includes additional attributes to enhance asset representation and enable an authentication mechanism to validate device authenticity. A detailed description of these additional attributes can be found in Table 2.

**Table 2:** Proposed metadata.

Sr #	Attribute	Description
1	Owner	EOA of the Owner
2	token_ID_	Token ID of the Owner
3	U_ID_	User Identification
4	DID	Smart Device Identification
5	FogID	Fog Device Identification
6	T	Time Stamp
7	∆T	Change in Time duration

In the proposed expansion in the ERC721 standard, devised attributes such as Owner (address) and token ID are defined, and their associated proposed metadata is utilized to validate ownership for managing resources. In order to represent user identification and device identification, UID and DID attributes were created, respectively. Additionally, the Fog_ID_ parameter is utilized for identifying fog nodes, and T and ∆T represent the block timestamp and time change for the blockstamp to prevent replay or spoofing attacks. The definitions and descriptions of these features are provided in Table 2 for enhanced comprehension.

### Components and mechanisms of the architecture

The proposed expansion has incorporated smart contracts (SCs) that are designed to enhance its versatility in accommodating various cyber-physical systems (CPSs) within smart city architecture. The modular components have been crafted in a manner that enables seamless integration into diverse settings such as smart homes, smart hospitals, smart supply chains, smart industries, smart cars, and other CPSs. The key components include the proposed smart contracts, the system owner (admin), the end-user, the fog device, and the IoT-enabled smart device. These components synergistically work together to achieve the objectives of the proposed expansion.

Table 3 outlines the interfaces and their respective components, including functions and events. The primary functions were developed within the proposed SC. To allow for customization, the OpenZeppelin Contracts library was employed to import ERC721 and integrate it seamlessly into the expanded NFT framework^33^. The components and mechanisms of the proposed NFT protocol have been presented with algorithms and pseudo-codes for clear understanding.

**Table 3:** Components and functions of the proposed NFT architecture.

*Functions & Events to add/Del and check the No. of Admins NFT EOAs*	function approve(address _approved, uint256 _tokenId) external payable;
	event AdminAdded(address indexed newAdmin, address indexed addingAdmin);
	event AdminAlreadyExists(address indexed newAdmin, address indexed sender);
	function No_ofAdmins() external view returns (uint256);
	function adminAdd() external view returns (address[] memory);
	function delAdmin (address admin) external;
	event AdminDeleted(address indexed newAdmin, address indexed deletingAdmin);
*Functions & Events to Add/Del/Map devices (IoT, Fog)*	function DeviceFogMapping(address fog, address device) external;
	event FogDeviceMappingAdded(address indexed fog, address indexed device, address indexed addingAdmin);
	event FogDeviceAllMappingDeleted(address indexed fog, address indexed deletingAdmin);
	event DeviceDoesnotExist(address indexed device, address indexed fog, address indexed sender);
	function delDev(address fog) external;
*Functions & Events to add/Del/Map Users with Smart devices*	function UserDeviceMapping(address user, address device,address fog) external;
	event UserDeviceAllMappingDeleted(address indexed user, address indexed deletingAdmin);
	event UserDeviceMappingAdded(address indexed user, address indexed device, address addingAdmin, address indexed fog);
	function delUser(address user) external;
*Functions & Events to check balance and owner of a token*	function balanceOf(address _owner) external view returns (uint256);
	function ownerOf(uint256 _tokenId) external view returns (address);
	function tokens_Issued()public view returns (Token[] memory);
*Minting Functions & Events for User and devices Authentication Mechanism*	function mintNFT(address device, address fog) external;
	event Authenticated(address indexed user, address indexed device, address indexed fog);
	event NotAuthenticated(address indexed user);
	event InvalidUser(address indexed device, address indexed fog, address indexed sender);
	event TokenCreated(bytes32 indexed _tokenID, address indexed User, address device, address indexed fog, uint256 timestamp);

The pseudo-code steps shown in Algorithm 1 depict the initialization of the proposed parameters and definitions of the components during the deployment of the SC. It shows the initialization of the Resource Owner/Admin, the only entity that can initiate the smart contract approved with initial proposed *approve* operators, i.e., token identification (token_id_) with an externally owned account (EOA). It is defined in the devised constructor to ensure the contract’s confidentiality, availability, and authorization to own and execute by this ID or otherwise reject the initialization. Once initiated, only the owner can update/add/delete and call the functions. Furthermore, lists and structs for admins, tokens, devices, and mapping functions have been devised for asset representation in the proposed mechanism.

**Algorithm 1:**
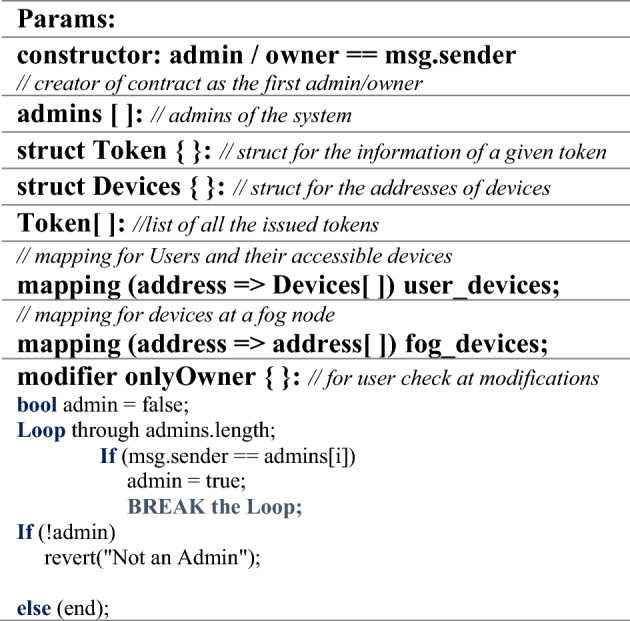
Initialization of Proposed NFT Params and Components Definition.

The metadata in Tables 2 and 3 includes the transaction payload in terms of NFT-based EOAs that employ the list of operations as depicted in the below-mentioned algorithms.

**Figure 5:**
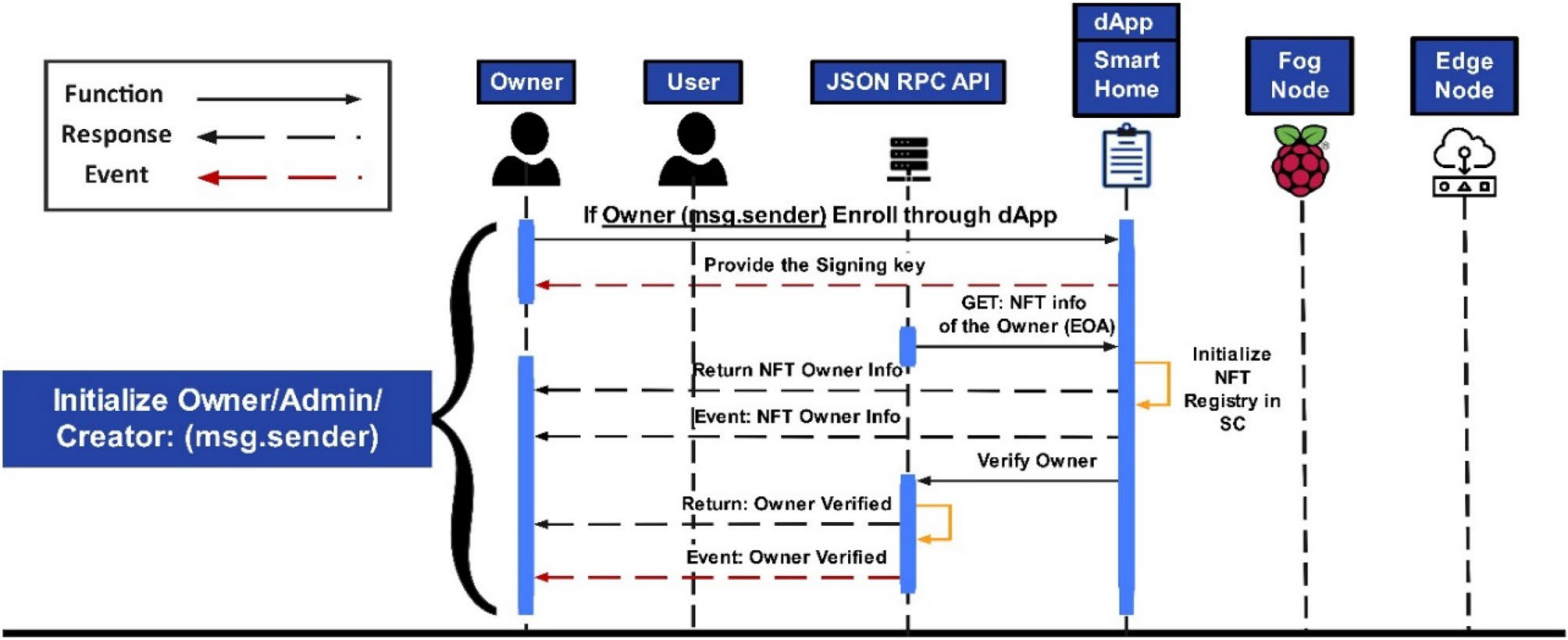
Sequence diagram of session connections for initializing owner representation.

Depicted in Fig. 5 is the process of initializing the owner and the secure session with the SC. The sequence diagram shows that the owner is the only admin or creator of the SC and will only initiate communication sessions for authorized access or will be rejected otherwise. It provides the owner the authorization to access a particular SC for execution in the proposed NFT architecture. Once verified, the owner provides the public and private key pair, which would initiate requesting the NFT info to generate the NFT for the owner. Once Token_ID_ is assigned, the information will be saved in the NFT registry, and details will be returned to the owner with an event. As aforementioned, the SC functions would be deployed based on the proposed mechanism of the ERC-721-IoT standard by the resource owner, who will be an admin in this context. Table 3 presents the functions that have been deployed.

Once the owner initializes the smart contract (SC), the assets must be verified. It would initiate the mapping function as a next step to associate the verified fog node with verified IoT assets, as depicted in Algorithm 2.

Events with the NFT EOA parameters (EOA_Fog_, EOA_Device_) will be generated and saved in the NFT registry. The next step is to initiate the IoT asset and fog device mapping, which would be initiated by the *DeviceFogMapping()* function that maps IoT devices with respective fog nodes with the NFT EOAs parameter (EOA_Fog_, EOA_Device_) as depicted in Algorithm 2.

**Algorithm 2:**
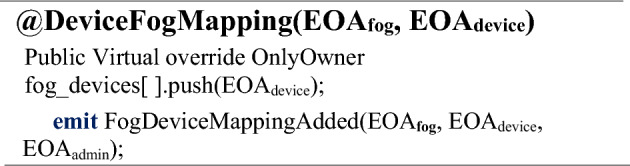
Assigning an IoT Node to Fog Node.

**Figure 6:**
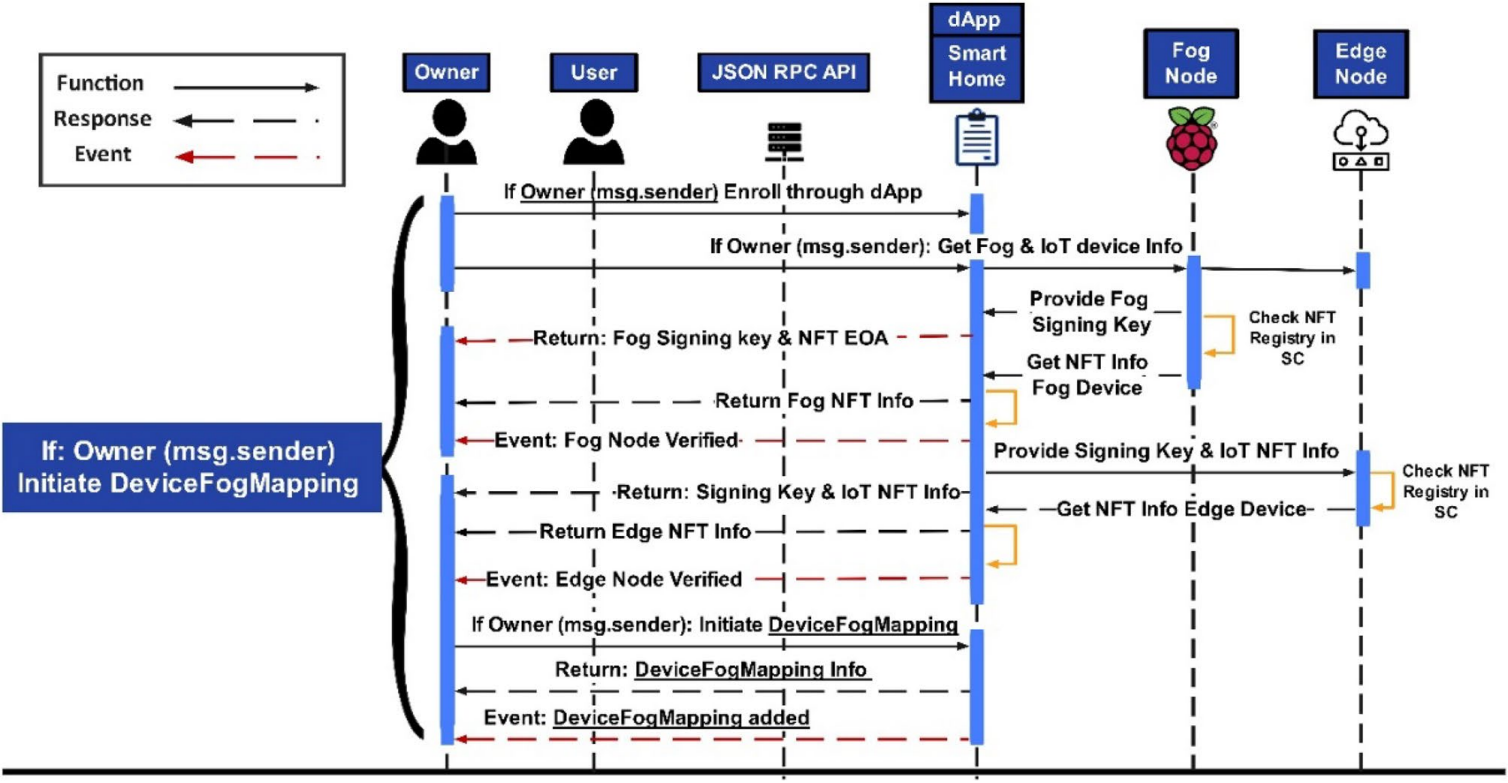
Sequence diagram of session connections for DeviceFogMapping representation by the owner.

Figure 6 depicts the sequence diagram of the owner initiating a secure session connection to authorize the fog device with the IoT-enabled smart device via mapping. The figure depicts the access authorization process only if the smart contract owner initializes the secure session with the smart contract or the access will be rejected otherwise. The owner queries the public and private key pair and NFT EOA information of the fog device in the first step. Once verified, the same procedure will be followed for the IoT assets in the second step. The *DeviceFogMapping()* function initiates that maps IoT devices with respective fog nodes with the NFT EOAs parameter (*EOA*_*Fog*_, *EOA*_*Device*_), and details are returned to the owner with events, as depicted.

The next step is to assign the mapped devices allotted to a user who can subsequently access them within the corresponding Cyber-Physical System (CPS). The owner gets the verified user (*EOA*_*User*_) and DeviceFogMapping (*EOA*_*Fog*_, *EOA*_*Device*_) information, and if the information is matched, the *UserDeviceMapping()* function will be initialized. This function would map the user with the fog and the IoT-enabled smart device via mapping to provide access to these devices once the authentication phase competes in the next step. At this point, the *UserDeviceMapping()* maps the user with respective fog and the IoT-enabled smart device with the NFT EOA parameters (EOA_*Fog*_, EOA_*Device*_), as depicted in Algorithm 3. Events with the NFT EOAs (*EOA*_*User*_*, EOA*_*Fog*_*, EOA*_*Device*_) will be generated and saved in the NFT registry. The algorithm shows the pseudo-code for the mapping process flow of the *UserDeviceFogMapping()* function. The User NFT EOAs parameter (*EOA*_*User*_) is assigned to the respective fog node. The fog node must have an IoT asset assigned to assign the user, or the request will be denied otherwise, as depicted in Algorithm 3.

**Algorithm 3:**
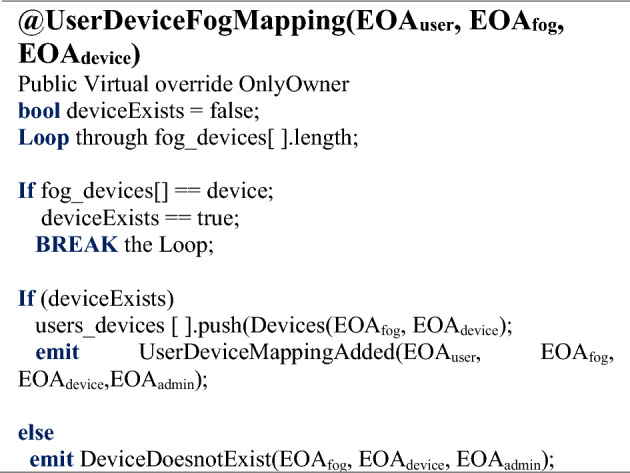
Assigning a User to a Fog Node with an assigned IoT Node.

**Figure 7:**
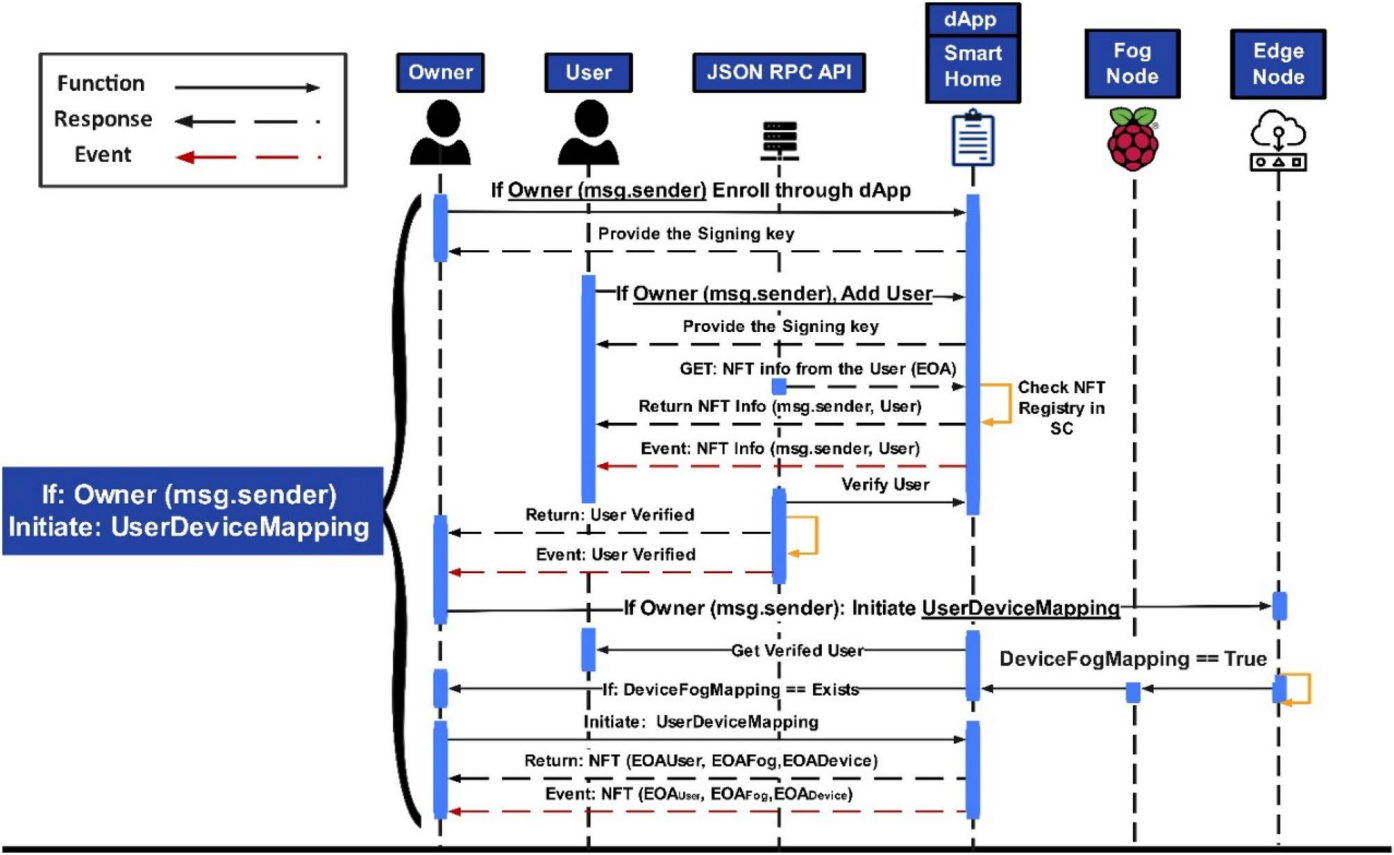
Sequence diagram of session connections for UserDeviceMapping representation.

Figure 7 depicts the owner initiating a secure session connection to add the user with its assigned NFT-based EOA and provides the public and private key pair, which, once verified, would initiate to request for the NFT info for the user. Once Token_ID_ is assigned, the information will be saved in the NFT registry, and details will be returned to the owner with an event. ﻿Once the IoT-enabled smart devices are mapped with the fog device and the respective user, the NFT minting function must be triggered to authenticate users to access the devices.

#### Minting and authentication mechanism

Algorithm 4 shows the *mintNFT()* function, which generates the user NFT (User_NFT_) to represent an authentication access token for the user to access the devices and for the authentication process every time the user accesses the nodes assigned. It is the final step where the authentication process will trigger by checking the EOA_*User*_, EOA_*Fog*_, and EOA_*Device*_ in the respective lists, as shown in Algorithm 3. An NFT for the user (User_NFT_) will be generated utilizing the SHAIII encryption protocol, which utilizes an authenticated encryption system as presented in this algorithm. It will perform authentication of assets by mapping the NFT-based Externally Owned Accounts (EOAs) of the users, fog, and IoT devices. If the mapping is successful, the assets will be authenticated, and if it fails, the system will reject the authentication request. The generated NFT_Id_ will be a unique identification access code used for user authentication whenever the user wants to access the devices, as depicted in Algorithm 4.

**Algorithm 4:**
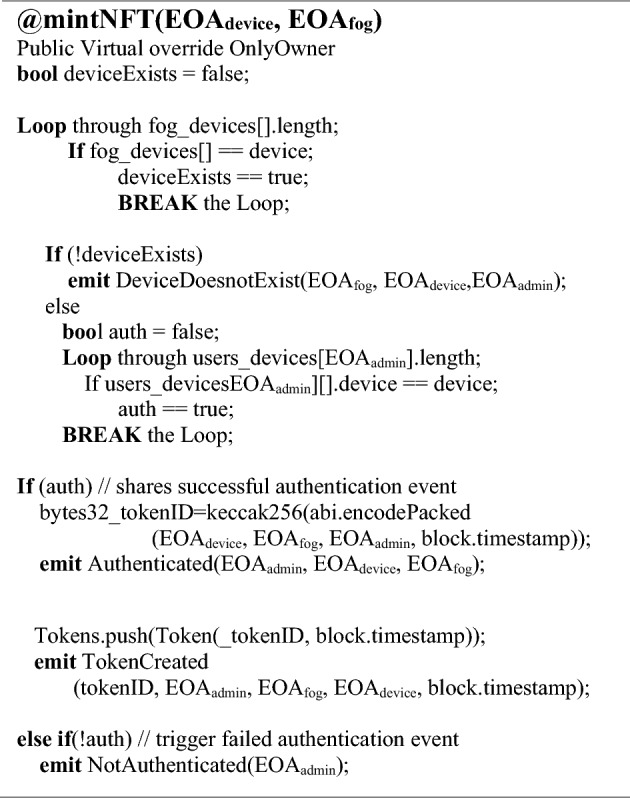
Mint Function to Create NFTs for UserFogDevice Authentication.

**Figure 8:**
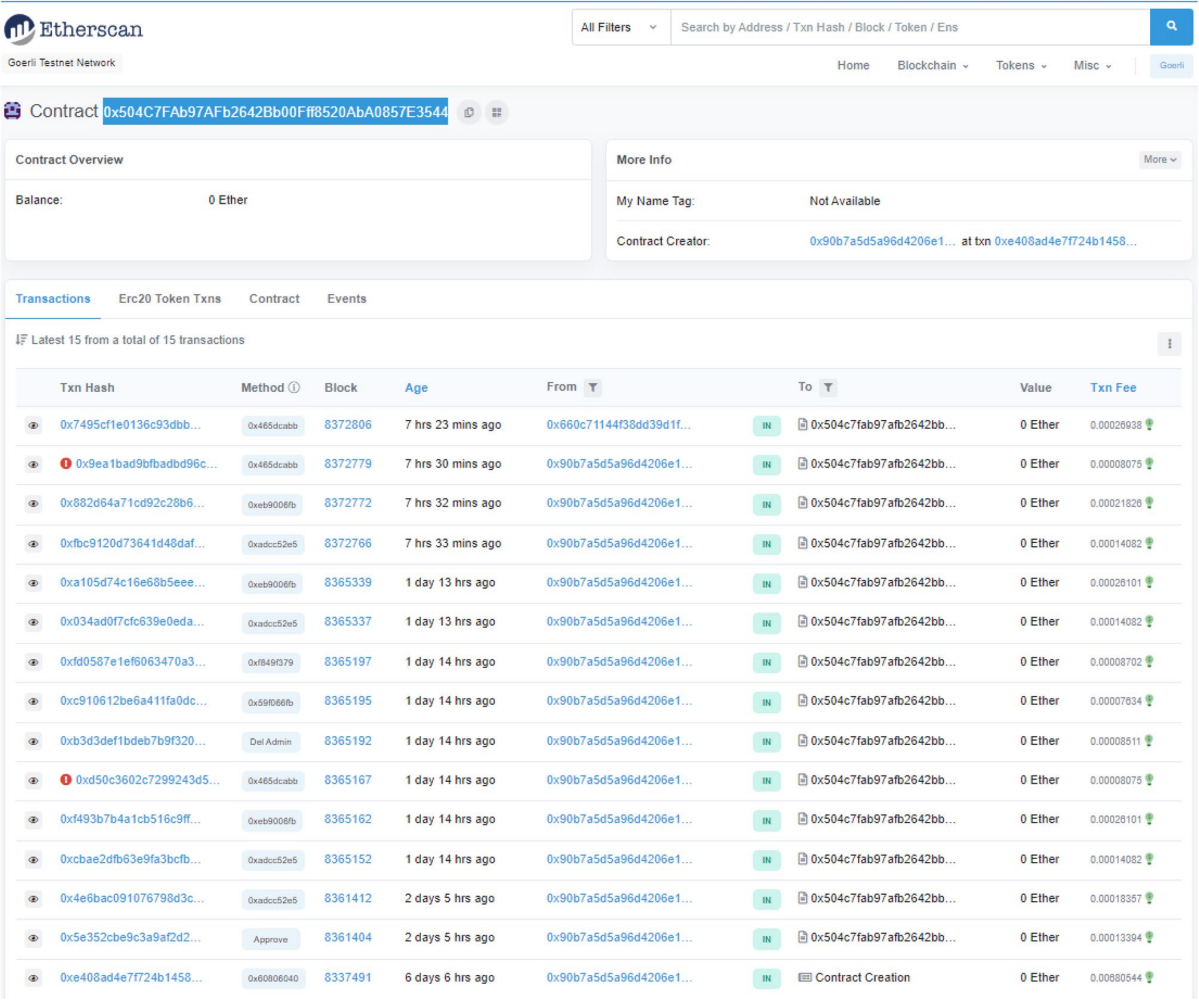
Posted transactions of NFT-based Architecture.

**Figure 9:**
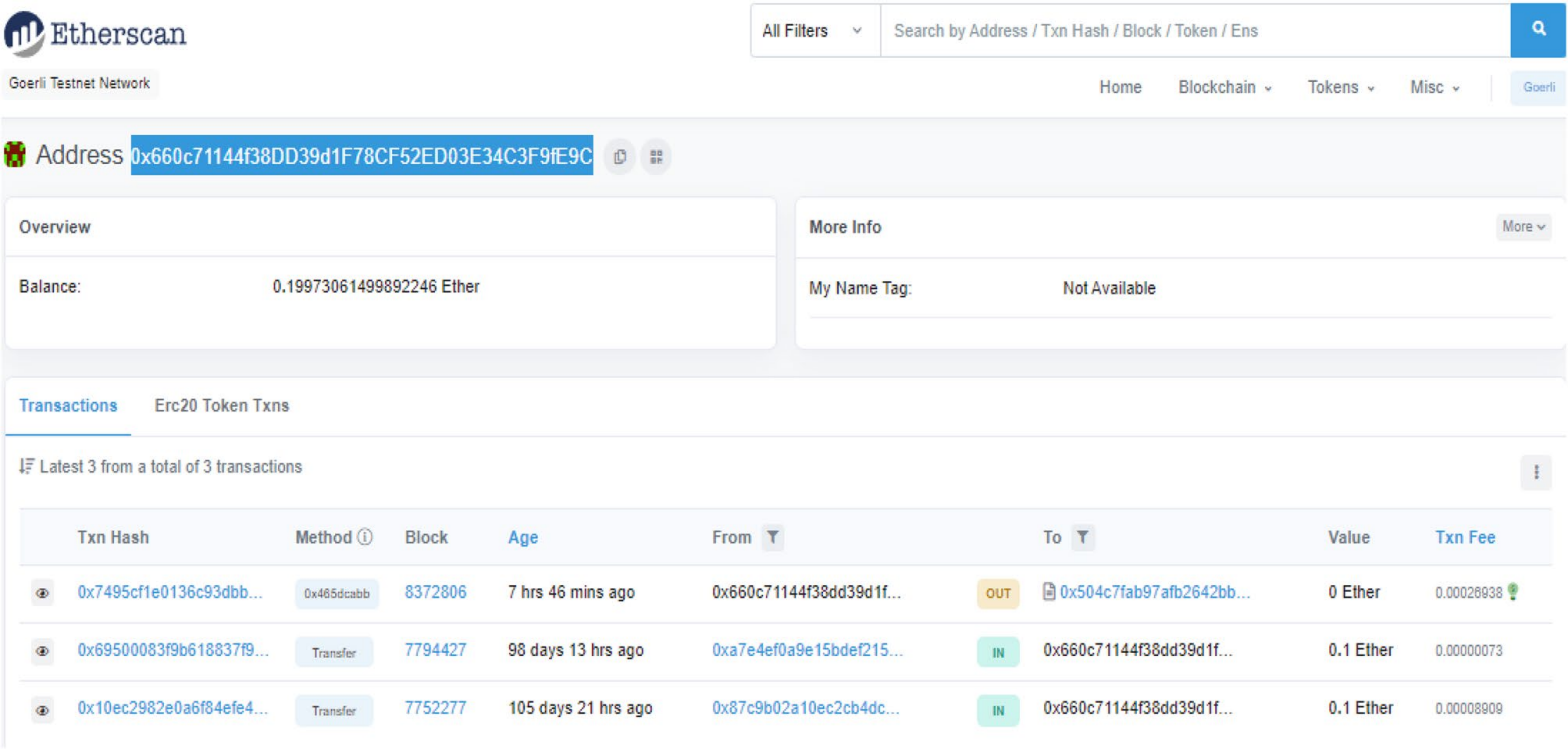
Authorized User transaction of NFT-based Architecture.

Figure 8 graphically represents the posted transactions of the proposed NFT architecture on the Goerli testnet while it also shows the failed transactions at transactions number 6 and 14 starting from the bottom. These transactions were rejected as authentication initialization was carried out using a different EOA_*User*_ which was not mapped and authorized. It resulted in a failed transaction hence proving the deployed mechanism. However, the NFT-based EOAs resulted in a successful transaction through the authorized user’s EOA_*User*_: 0 × 660c71144f38DD39d1F78CF52ED03E34C3F9fE9C since the authentication request was successful due to the mapping of NFT-based externally owned accounts (EOAs) belonging to users, fog nodes, and IoT devices as shown in Fig. 9.

**Figure 10:**
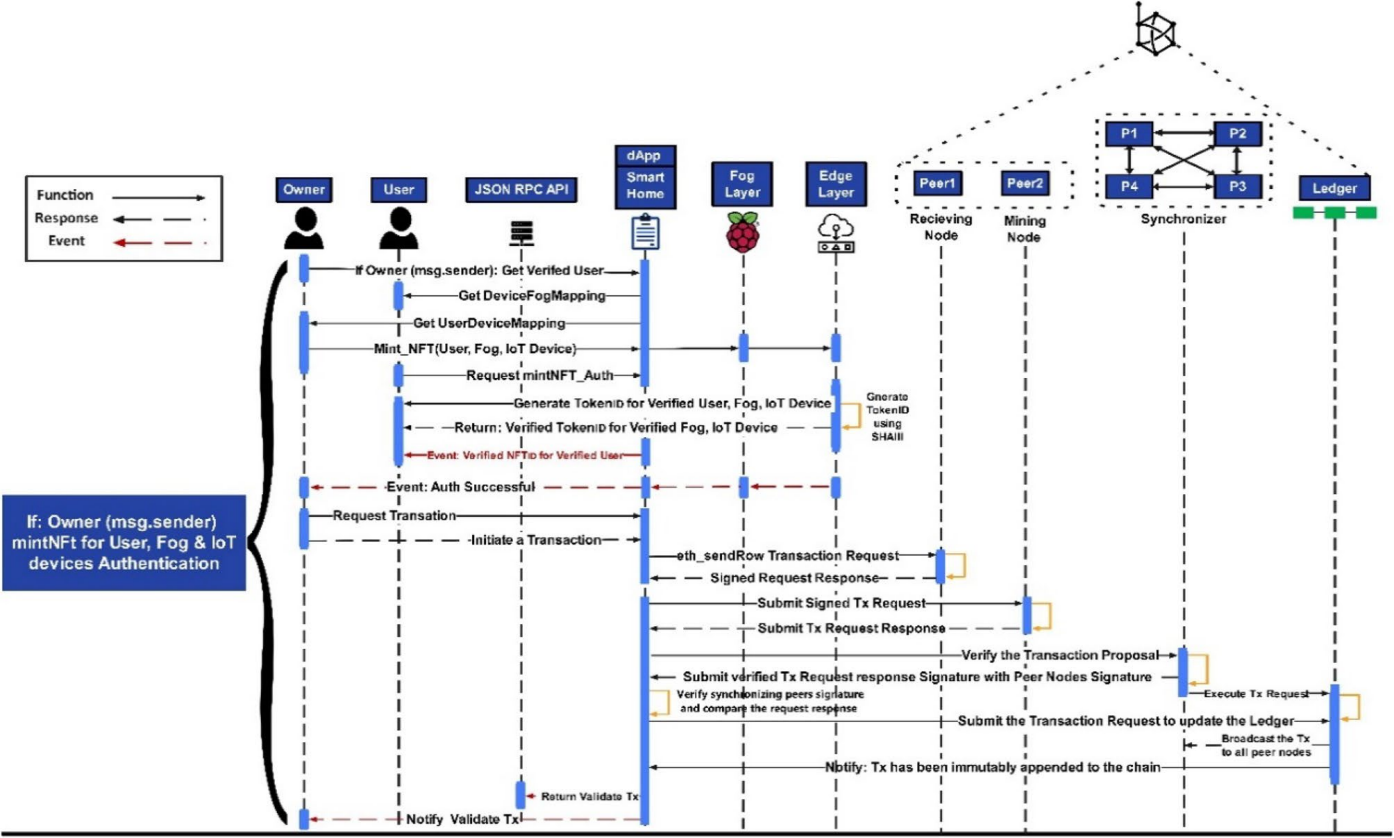
Sequence diagram of session connections for mintNFT_Auth representation.

Figure 10 depicts the mapped user initiating a secure session connection to authenticate and generate NFT for user authentication. A complete process of posting transactions has been shown in the sequence diagram, so a complete blockchain working may be presented. The pseudo-code in Algorithm 4 shows the function to verify if the IoT nodes are assigned to the fog nodes and the user. The request to authenticate the user will be denied if no device is detected in the fog list. Upon NFT authentication, the node signs it with the user’s account public key (User_PK_).

$$- {\text{ NFT}}_{{{\text{pass}}}} = {\text{Pass}}_{{{\text{User}}}} \left( {{\text{User}}_{{{\text{NFT}}}} } \right),\;{\text{User}}_{{{\text{NFT}}}} {\text{whereas}},\;{\text{User}}_{{{\text{NFT}}}} = ({\text{UID}},\;{\text{T}},\Delta {\text{T}},\;{\text{EOA}}_{{{\text{User}}}} ,\;{\text{EOA}}_{{{\text{Device}}}} ,\;{\text{EOA}}_{{{\text{Fog}},}} {\text{User}}_{{{\text{PK}}}} ).$$

The user signs the token with the account’s private key (User_IK_), as shown below, and the user is authenticated to access the mapped devices.

$$- {\text{ NFT}}_{{{\text{pass}}}} = {\text{ Pass}}_{{{\text{User}}}} \left( {{\text{User}}_{{{\text{NFT}}}} } \right)\;{\text{whereas}},\;{\text{User}}_{{{\text{NFT}}}} = \, \left( {{\text{Token}}_{{{\text{Id}}}} ,\;{\text{T}},\;\Delta {\text{T}},\;{\text{EOA}}_{{{\text{User}}}} ,\;{\text{EOA}}_{{{\text{Device}}}} ,\;{\text{EOA}}_{{{\text{Fog}}}} ,\;{\text{User}}_{{{\text{IK}}}} } \right).$$

The NFT_Pass_ represents the user’s non-fungible token generated by incorporating the user’s token, block timestamp, and change in block timestamp together with the Ethereum account addresses of the users, fog nodes, and IoT devices, and the user’s EOA private key. In order to facilitate user access to the assigned nodes, an authentication access token is generated by the system. This token is used to verify the user's identity during subsequent authentication attempts when accessing the devices. An inventive approach of *call()* methods has been devised to retrieve the status of assets in the NFT registry, the smart contract can be queried, and this action incurs no transaction cost in Ether or Gewi. It demonstrates the proposed architecture's efficiency in terms of time complexity. Only the owner can perform the call operations; otherwise, the request will be rejected. The events will be emitted once a specific operation has been performed. The smart devices (SDs) define the NFT-based EOAs mapping mechanism with a particular user EOA, ensuring security services as discussed in “Security Services” section.

Once a successful event has been generated, the owner requests to initialize the transaction to append the blockchain, as shown in Fig. 10. The transaction request is generated to a p2p network of receiving nodes, which drives the signed request response. The transaction with a signed request-response is submitted to the p2p network of mining nodes which verifies the transaction and submits the transaction proposal to a synchronizer p2p network. It verifies the synchronizing peers’ signatures and compares the request response to verify it. Once the response is verified, it is posted to the ledger.

After the transaction record has been appended to the ledger, the updated transaction request response is broadcasted to all the peers to synchronize the transaction and append the chain to attain immutability. The validated response is returned and notified to the owner via an event. The research's source code is readily available for public access on GitHub^34^ and can be conveniently deployed on the Goerli testnet.

## Results and discussion

### Security services

The literature review reveals that none of the proposals have taken into consideration security services such as confidentiality, integrity, and availability (CIA) and have heavily relied on the default security mechanisms. Some rely only on the basic consensus mechanism, while these proposals achieve integrity by implementing the encryption protocol, as shown in Table five. However, the proposed NFT architecture achieves security services (CIA) and authorization as presented in Table 4.

**Table 4:** Security services in the proposed NFT architecture.

Security Services	Protection
Confidentiality	Achieved by devising a constructor using SC
Integrity	Implements Encryption Protocol—SHA III
Availability	Achieved by devising a constructor using SC
Traceability	Achieved using Hyperledger Besu P2P Synchronizer Network
Authorization	Achieved by devising a modifier for “OnlyOwner” using SC

#### Confidentiality and availability

As shown in Algorithm 1, to achieve the security service, a constructor has been devised to ensure the smart contract’s (SC) confidentiality and availability, which would only allow the Creator/Admin/Owner to own the smart contract. The owner will be able to execute the functions by its ID or initialization will be rejected. Once initiated, only the owner can update/add/delete/call other admins, users, IoT assets, and fog devices. This property helped achieve confidentiality, while the availability of SC assets is restricted only to the owner who does not allow the resource availability to anyone apart from owners as shown in Table 4.

Figure 8 graphically represents the failed transactions of the proposed NFT architecture on the Goerli testnet as authentication initialization was carried out using non-owner EOA. Similarly, the NFT-based EOAs resulted in a successful transaction through the authorized user’s EOA_*User*_ since the NFT-based externally owned accounts (EOAs) of the users, fog, and IoT devices were mapped resulting in a successful authentication request as shown in Fig. 9. This shows that the proposed NFT architecture accomplishes the confidentiality and availability of the assets efficiently.

#### Authorization

Algorithm 1 further depicts a modifier devised and defined as “OnlyOwner.” It adds another layer of security as the owner (msg.sender) defined in the contract’s constructor will be registered as the only owner who can log into the smart contract. It accesses all the smart contract functions for adding users and smart devices. It ensures only approved (*approve()*) admin/onlyOwner as an authorized entity to initiate all the functions requests. Otherwise, the access will be denied, which fulfills the need for the authorized user, such as the owner, to achieve authorization in smart city architecture as shown in Table 4.

Figure 8 graphically represents the failed transactions of the proposed NFT architecture on the Goerli testnet as authentication initialization was carried out using a different EOA_*User*_ which was not authorized. It resulted in a failed transaction hence proving the deployed mechanism. Similarly, the NFT-based EOAs resulted in a successful transaction through the user’s EOA_*User*_ as it was authorized by the owner resulting in a successful authentication request as shown in Fig. 9.

#### Integrity

The presented research has integrated the proposed architecture for robust authentication, utilizing the functionality of the SHA-III encryption protocol as illustrated in Algorithm 4. The *mintNFT* function at the blockchain layer has been deployed to execute the proposed authentication mechanism. Other potential uses of the function, such as an authenticated encryption system, would benefit from faster hashing in the proposed architecture. As illustrated in Table 4, the process utilizes the SHA-III algorithm to generate the NFT Token_ID_ for the user, which will be a unique identification code used for user authentication whenever the user needs to access the devices.

### Validation of proposed NFT architecture

After the deployment of the testbed, it was necessary to validate the smart contract methods that utilize proposed NFTs by comparing the amount of gas consumed during transaction execution between private deployment on Besu and deployment on the Goerli testnet platform. The Ethereum blockchain is powered by a cryptocurrency called ether (ETH), which is divided into smaller fractions known as Gwei. Gas is the term used to describe the cost of executing operations that modify data on the blockchain. In the course of executing a decentralized application (dApp), including smart contracts, resources are allocated by consuming gas. The gas limit required for executing a lightweight implementation of a dApp would be lower, thus reducing the work required to complete a transaction using ETH (Ether) via a smart contract.

**Figure 11:**
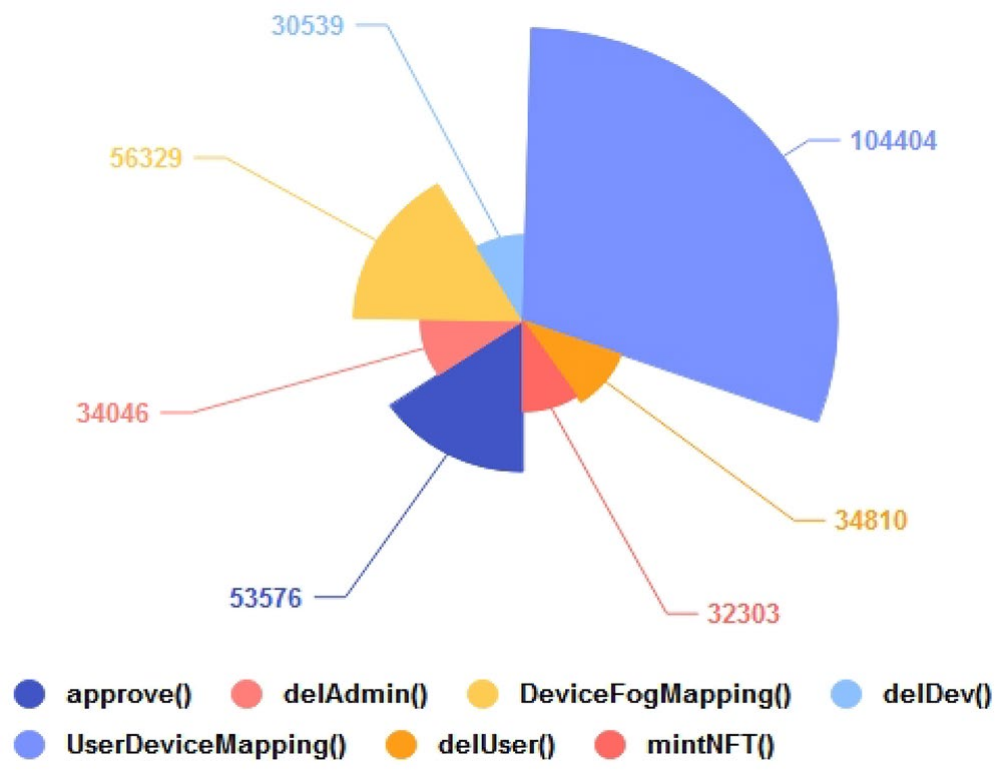
Proposed NFT execution costs ~ Goerli Testnet.

Conversely, a more gas-intensive implementation would be inefficient. To evaluate the gas consumption of the proposed NFT functions during deployment, their execution cost was measured, allowing for a better understanding of the cost of each function. Figure 11 illustrates the execution cost of the primary components of the proposed NFT on the Goerli testnet. As expected, the *UserDeviceMapping()* function consumed the most gas due to the mapping of users and devices. Additionally, the *DeviceFogMapping()* function, which maps the respective fog device to the IoT nodes, consumed a significant amount of gas. In contrast, the average gas consumption of other functions, i.e., *approve(), delAdmin(), delDev(), delUser(),* and *mint(),* was nearly identical.

**Figure 12:**
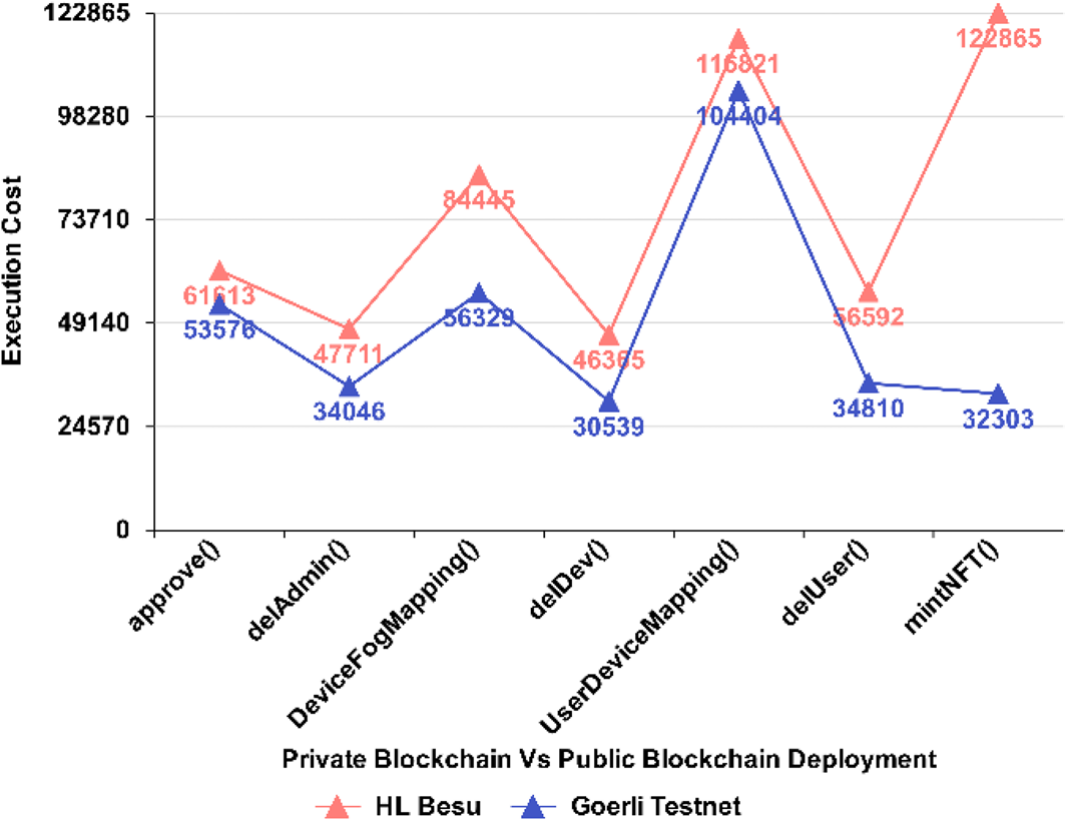
Cost comparison of the proposed NFT architecture over HL Besu ~ Goerli Testnet.

Figure 12 shows the execution cost comparison of proposed NFT components, where deployment on a private blockchain network i.e., HL Besu shows high execution cost compared to the deployment on a public testnet which is Georli in this case. Based on the results, it is apparent that the execution costs of all functions have increased, with the *mint()* function consuming more Gas. This is not surprising, given that the encryption and authentication of users and devices are involved. The *DeviceFogMapping() and UserDeviceMapping()* functions also consumed more gas on a private blockchain network. Likewise, other components such as *approve(), delAdmin(), delDev(), and delUser()* functions have consumed a comparable amount of Gas on average but an increased execution cost has been observed which shows that the proposed NFT architecture will perform better on public blockchain networks however the increased execution cost over private deployment may have increased execution cost because of the block size which increases or decreases following the network demand.

### Efficiency performance

The review of the literature makes it evident that the digitization of the IoT-enabled smart assets and authentication mechanisms utilizing NFTs is lacking; hence, comparing the proposed architecture would not be possible. However, as presented in Table 5, a solution based on PUF-based NFT for linking the IoT assets has been proposed in^15^, utilizing the hardware modifications in the IoT assets as in the use of physically unclonable functions (PUFs). It is used for IoT asset identification. Since the solution depends on binding the NFT with PUFs to identify an IoT asset and authentication mechanism, the results show high latency in terms of device initialization time. The functions and components in the proposed architecture have been observed to have consumed more gas to perform the transactions to append the data on the blockchain^15^.

**Table 5:** Comparison with related cutting-edge blockchain-based authentication mechanisms.

Ref	Year proposed	Blockchain (BC) platform	Consensus mechanism	NFT	Time complexity	CIA
^18^	2020	Public BC (Eth)	PoW	✗	O(n)	I
^35^	2021	Public BC (Eth)	PoW	✗	O(m ∗ n)	I
^36^	2021	Private BC (HL Fabric)	PBFT	✗	O(n^2^)	I
^19^	2018	Public BC (Eth)	PoW	✗	O(n)	A
^20^	2020	Public BC (Namecoin)	PoW	✗	O(n)	I
^21^	2018	Public BC (Eth)	PoW	✗	O(n)	CI
^22^	2018	Public BC (Eth)	PoW	✗	O(n)	✓
^37^	2017	Public BC (Eth)	PoW	✗	O(n)	✗
^27^	2021	Public BC (Eth) / Private BC (HL Fabric)	PoW/PBFT	✗	O(n)	I
^15^	2021	Public BC (Eth)	PoW	✓	O(n)	I
Proposed NFT architecture	2022	Private BC (HL Besu)/Goerli Testnet	IBFT 2.0	✓	O(n)	✓

**Figure 13:**
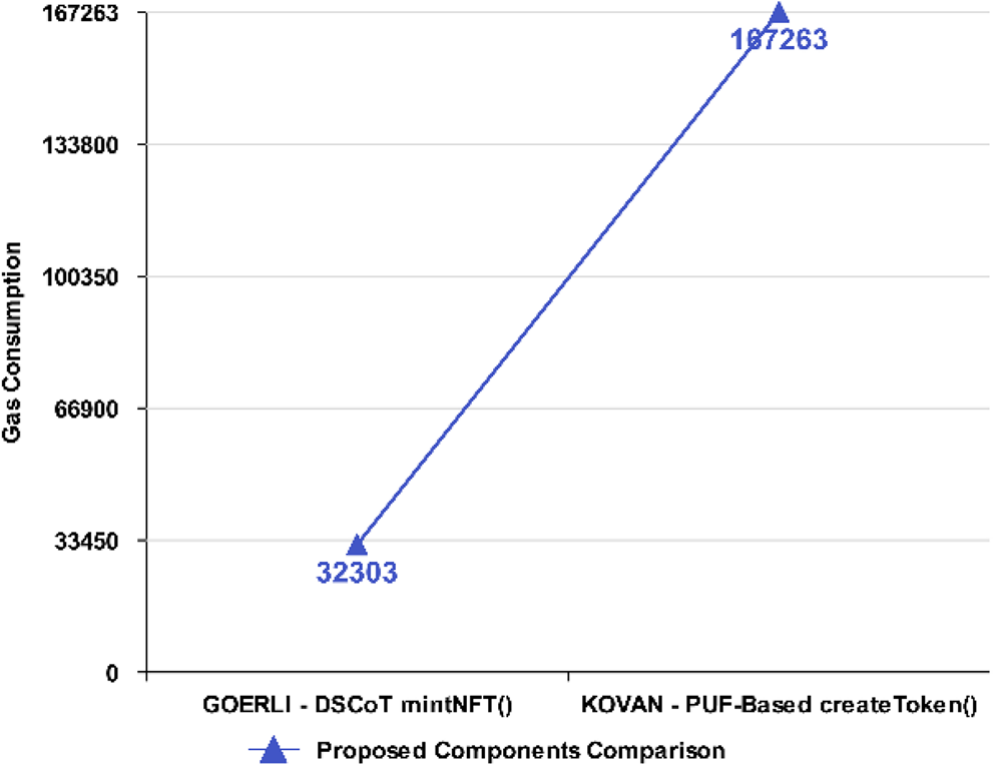
Execution Cost ~ Proposed NFT mint.

A comparison of execution rate in terms of Gas consumption for the proposed NFT architecture was evaluated by its Gas consumption in contrast to the PUF-based NFT solution as presented in Fig. 13, which depicts a considerably low gas consumption for main minting functions. Evidently, it shows proposed NFT *mintNFT()* function is more efficient as it consumes lower Gas (32,303) than the PUF-based NFT *createToken()* function, which consumes more Gas (167,263) comparatively.

**Figure 14:**
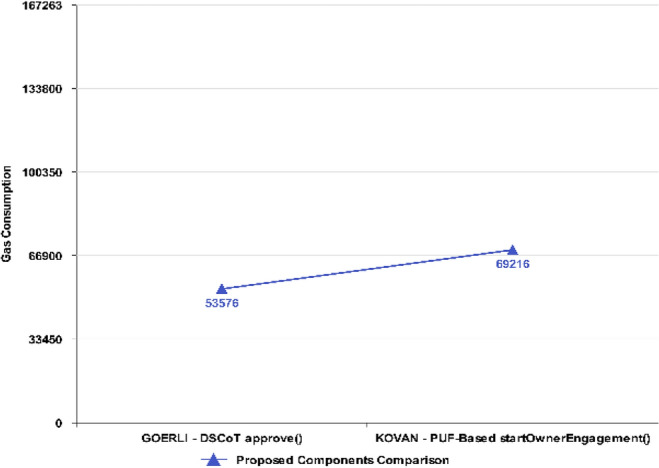
Execution Cost ~ approve.

The *approve()* function in Fig. 14 also shows considerably low gas consumption (53,576) for the proposed NFT in contrast to the *startOwnerEngagement()* function, which comparatively consumes more Gas (69,216).

**Figure 15:**
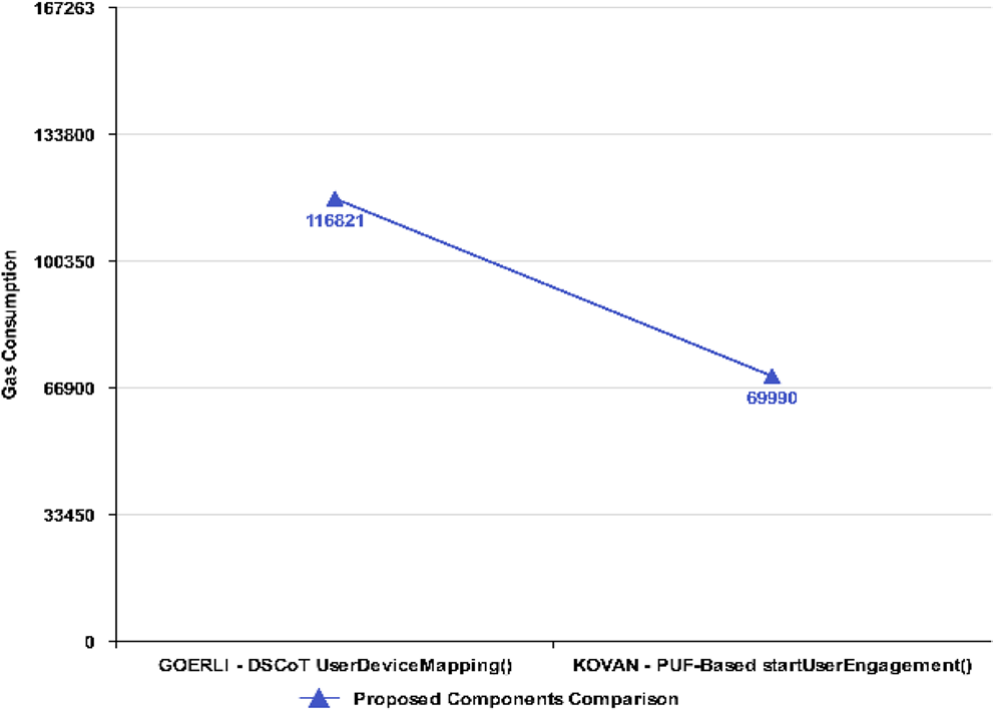
Execution Cost ~ UserDeviceMapping.

The *UserDeviceMapping()* function in Fig. 15, on the other hand, consumes low gas consumption (116,821) but comparatively depicts more than the PUF-based NFT’s (69,990) *startUserEngagement()* function. The rise has been observed due to the User and Device Mapping that takes place at this stage simultaneously in the proposed mechanism, while the startUserEngagement() in PUF-based NFT consumes low gas because it is used to engage the user only.

**Figure 16:**
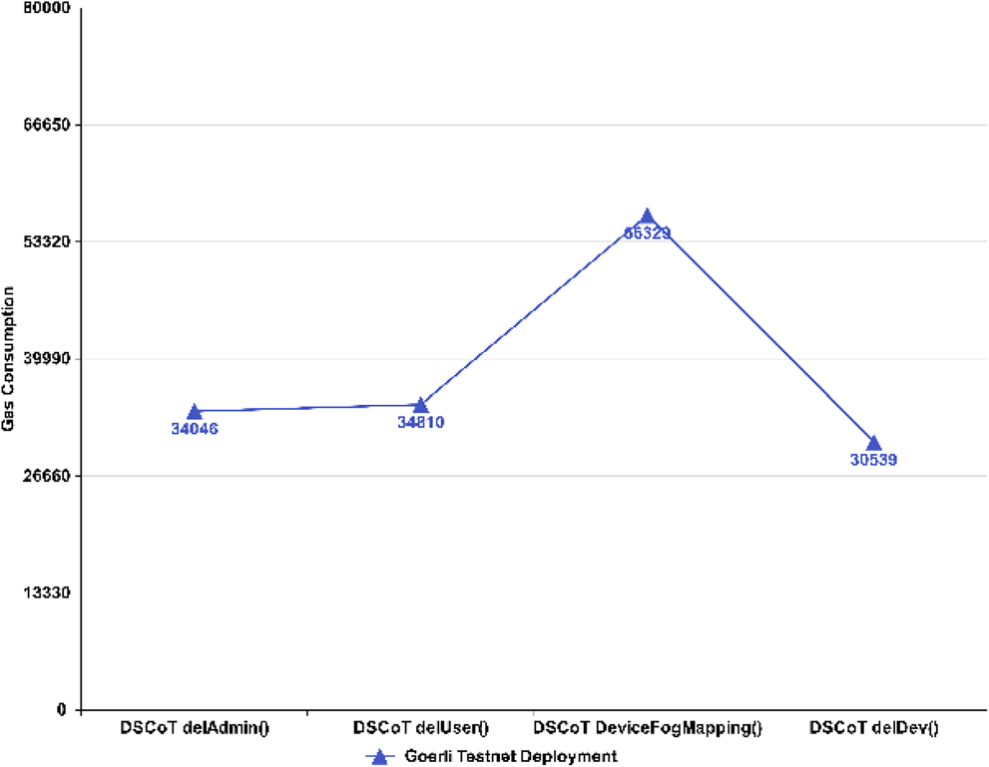
Execution Cost ~ call to NFT registry.

Figure 16 shows the gas consumption for proposed functions that have been created to check the status of the NFT registry with considerably low gas consumption. These functions could not be compared with other functions of PUF-based NFT solutions as they could not be compared in terms of their utilization and functionality; however, the depicted functions in the proposed NFT remain efficient in terms of low gas consumption.

**Figure 17:**
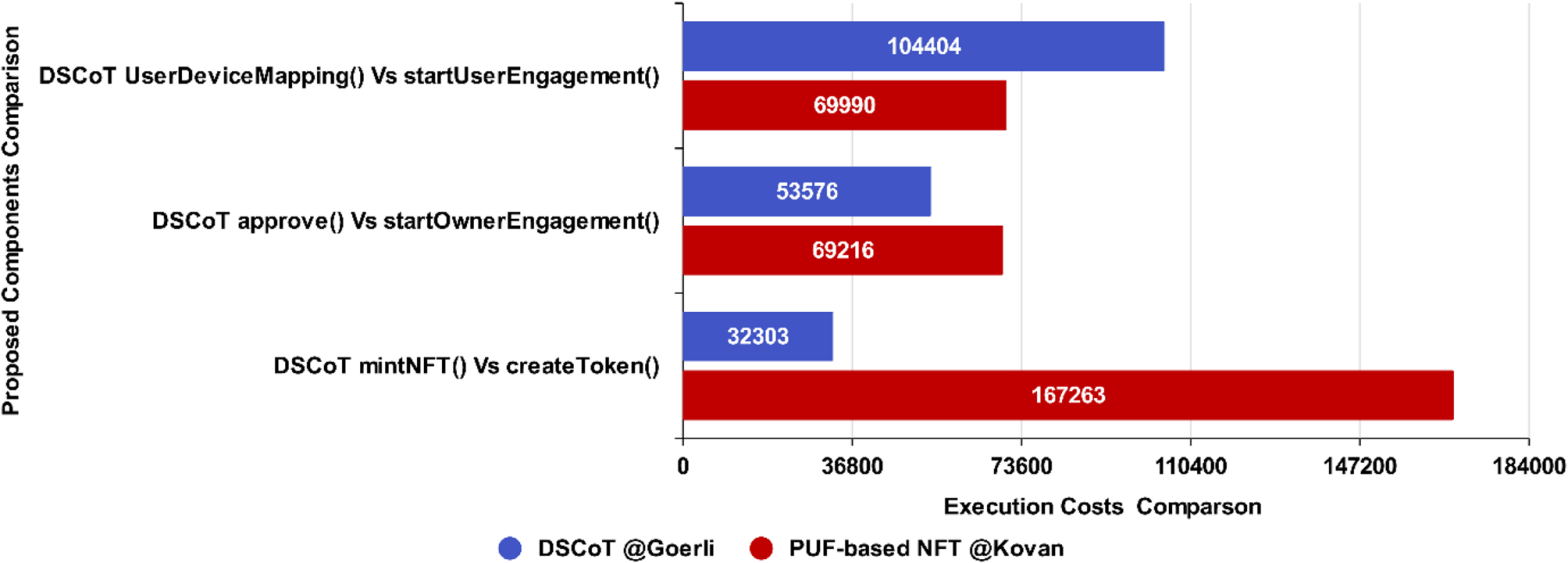
Execution costs of proposed NFT vs. PUF-based NFT over Testnets.

Figure 17 provides a broader graphical view of the execution cost of proposed components in the proposed NFT (*BLUE*) and PUF-based NFT (*RED*), which depicts a considerably low gas consumption for the proposed NFT architecture. As shown in the graph, it is evident that the proposed *mintNFT()* function is significantly 81% more cost-efficient than the *createToken()* function. Similarly, the proposed *approve()* function is approximately 23% more cost-efficient than the *startOwnerEngagement()* function. Furthermore, Table 5 compares cutting-edge BC-based solutions with the proposed NFT architecture, which explicitly implements the Hyperledger Besu to make the NFT-based architecture robust by employing the (IBFT 2.0 consensus mechanism and Goerli testnet that employs the Proof of Stake (*PoS*) consensus mechanism. At the same time, many of the existing solutions have been deployed on public blockchain networks that rely on default consensus mechanisms, which can lead to performance issues related to fault tolerance, decentralization, stability, and security. The private blockchain implementation attains consensus within hundreds of milliseconds, providing low latency. This property of the consensus mechanism is crucial for building blockchain-based IoT networks that provide low communication overheads and fault tolerance^38^.

In contrast, among all the BC-based solutions, only the authors in^15^ proposed an NFT extension and managed to employ an NFT-based solution for user and IoT-enabled smart device authentication mechanisms. However, the authors in^15^ utilize the PUFs for authentication and implement the default consensus mechanism of Proof of Work (*POW*) which does not look promising for CPSs because of the high energy consumption, communication overheads, low fault tolerance, and latency issues^15^. Apart from the proposed NFT architecture, none of the solutions manage to deploy all the features of security services (i.e., confidentiality, availability, and integrity), making it more robust in terms of security.

### Time complexity for latency

The suggested protocol for the NFT registry does not involve modifying data on the blockchain to counter-check the identity of proposed tasks and mechanisms. Instead, a novel approach to call methods has been devised to inquire about the state of assets in the registry by querying the smart contract. This approach does not alter any data on the chain and results in a reduction in transaction costs (measured in Ether/Gwei) while also being efficient in terms of time complexity. The latency results obtained on a private HL Besu deployment have been validated by the results obtained over the Goerli testnet as presented below.

**Figure 18:**
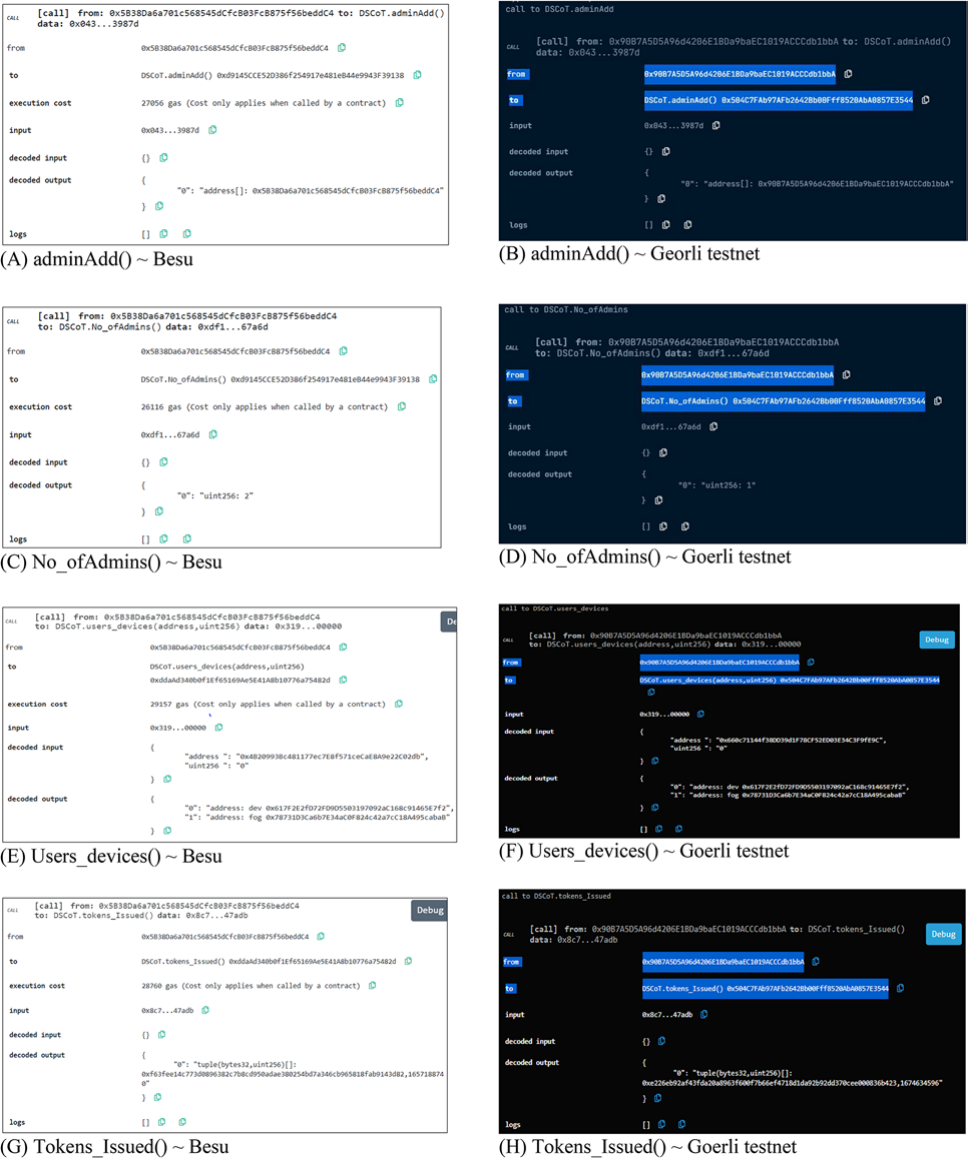
The time complexity of the proposed NFT architecture.

- In accordance with the proposed architecture, a call method named "*adminAdd()*" has been developed to discover the admin/owner addresses, as illustrated in Fig. 18A. The output of the method displays the admin NFT-based externally owned account (EOA) on Besu as "*0* × *5B38Da6a701c568545dCfcB03FcB875f56beddC4*."

  - The method (“*adminAdd()”)* was also validated over testnet deployment to find the admins/Owner addresses, as shown in Fig. 18B, which shows “0 × 90B7A5D5A96d4206E1BDa9baEC1019ACCCdb1bbA” as an admin NFT-based EOA.
- A call method has been designed, named "*No_ofAdmins()*", to ascertain the overall count of admins/owners in the system. Figure 18C indicates that there are "2" admin addresses present.
- The validation of the method was carried out through deployment on a testnet, to ascertain the total number of admins/Owners, as illustrated in as shown in Fig. 18D, which shows “1” admin address exists. The “*user_Devices_Add()* call method has been designed to find the total number of devices mapped to a specific user, as shown in Fig. 18E, which shows that two NFT-based EOAs exist, i.e., fog: “0 × 78731D3Ca6b7E34aC0F824c42a7cC18A495cabaB” and IoT device: “0 × 617F2E2fD72FD9D5503197092aC168c91465E7f2”.
  - The method was also validated through a testnet deployment to determine the total number of devices mapped to the user as shown in Fig. 18F, which reveals the existence of two NFT-based EOAs, i.e., “0 × 78731D3Ca6b7E34aC0F824c42a7cC18A495cabaB” for the fog “0 × 617F2E2fD72FD9D5503197092aC168c91465E7f2” for IoT device.
- In order to retrieve the total number of NFTs issued, a call method called "tokens_Issued()" was developed, as illustrated in Fig. 18G. The method retrieves the NFT access token, along with the block timestamp, which can be used for various purposes. The sample output in the figure shows the generation of an NFT access token with the hash value "*0xf63fee14c773d0896382c7b8cd950adae380254bd7a346cb965818fab9143d82*" and a timestamp of "*1,657,188,740*".
  - The method was also validated over testnet deployment to determine the total number of NFTs, as shown in Fig. 18H, which reveals “0xe226eb92af43fda20a8963f600f7b66ef4718d1da92b92dd370cee000836b423,1,674,634,596” generated NFT access token alongside a block timestamp.

In Hyperledger Besu, a private blockchain that is based on Ethereum, the duration of generating new blocks is influenced by the size of the block. Although transactions with transaction fees may experience delays, increasing transaction fees could potentially resolve this issue. Goerli testnet on the other hand is a community-based project, a completely open-source blockchain network that proved to be helpful as it is a valuable tool for testing and implementing blockchain-based decentralized apps. It was used for testing and validating the results obtained in a public test environment. After performing more than 500 calls to the functions previously discussed, we concluded that they were efficient. Moreover, the call methods created in the smart contract to obtain the asset status in the NFT registry were validated to not incur any gas fees for transactions. The proposed architecture has an O(n) time complexity in the case of 'n' IoT devices that need to be authenticated for identity.

## Conclusion

The cyber-physical systems in smart city architectures are prone to various adversaries. The present study proposes a distributed model based on blockchain tokenization. The proposed architecture introduces a novel approach for representing smart devices suggesting expansion in non-fungible tokens (NFTs) based on the ERC-721 standard, along with an authentication mechanism for the devices and their respective admin/owner and user. The main objective of the proposed architecture is to address adversarial issues that may arise in a distributed environment. Smart contracts based on the proposed NFT architecture were developed on private and testnet blockchain platforms, incorporating robust consensus mechanisms IBFT 2.0 and PoS, respectively. The proposed smart contracts comprise various components and procedures, each associated with the proposed NFT-based externally owned account (NFT_EOA_). In addition, robust security measures, including confidentiality, integrity, availability (CIA), and authorization, have been successfully integrated. To identify and authenticate users and devices, a mechanism was devised in the extended version of NFTs as unique, non-interchangeable identifying codes for each physical asset's ownership. The evaluation of the proposed functions and components has been conducted concerning their execution cost, efficiency, and time complexity, which yielded promising results. Comparatively, the Gas consumption for the proposed showed approximately ≈ 81%, while the proposed *approve()* was approximately ≈ 23% more efficient, respectively. Novel methods of call() functions have been developed to check the current condition or state of assets within the NFT registry without modifying any data on the chain. This innovative approach helps to reduce transaction costs (in Ether/Gewi) and improve the efficiency of the proposed architecture in regard to time complexity. In addition, the proposed NFT architecture will be used to create a digital representation of smart devices such as IoT and fog. The architecture proposed in this paper will be tested in use-case scenarios for smart houses and smart hospitals in the future to further validate its rigor, security services, and validation. Subsequently, the findings will be reported to demonstrate the efficacy and practicality of the proposed architecture.

## Data Availability

The authors did not use any external dataset for simulation. The simulations are performed through proposed Smart Contracts using Remix IDE which has built-in modules to deploy models to public and private blockchain ledgers. The source code has been made available on the GitHub repository^34^, however, the corresponding author may be contacted in case of further discussion.
